# *In silico* analysis of antibiotic-induced *Clostridium difficile* infection: Remediation techniques and biological adaptations

**DOI:** 10.1371/journal.pcbi.1006001

**Published:** 2018-02-16

**Authors:** Eric W. Jones, Jean M. Carlson

**Affiliations:** Department of Physics, University of California at Santa Barbara, Santa Barbara, California, United States of America; ETH Zurich, SWITZERLAND

## Abstract

In this paper we study antibiotic-induced *C. difficile* infection (CDI), caused by the toxin-producing *C. difficile* (CD), and implement clinically-inspired simulated treatments in a computational framework that synthesizes a generalized Lotka-Volterra (gLV) model with SIR modeling techniques. The gLV model uses parameters derived from an experimental mouse model, in which the mice are administered antibiotics and subsequently dosed with CD. We numerically identify which of the experimentally measured initial conditions are vulnerable to CD colonization, then formalize the notion of *CD susceptibility* analytically. We simulate fecal transplantation, a clinically successful treatment for CDI, and discover that both the transplant timing and transplant donor are relevant to the the efficacy of the treatment, a result which has clinical implications. We incorporate two nongeneric yet dangerous attributes of CD into the gLV model, sporulation and antibiotic-resistant mutation, and for each identify relevant SIR techniques that describe the desired attribute. Finally, we rely on the results of our framework to analyze an experimental study of fecal transplants in mice, and are able to explain observed experimental results, validate our simulated results, and suggest model-motivated experiments.

## Author summary

The burgeoning integration of big data and medicine is a portent of personalized healthcare. There is a need for accurate, predictive, and mechanistic models that can be relied upon to forecast the course of a disease, test treatments in-silico, and ultimately inform the doctor’s prescription. These models, still nascent, are buoyed by rich datasets available due to recent advances in experimental methods (e.g. 16S rRNA high-throughput sequencing); one such model, which we build upon in this paper, was developed by Stein *et al.* to predict the growth of the infectious *C. difficile* (CD) and 10 other microbial genera. In this paper we extend the existing model to capture clinical treatments and biologically relevant phenomena. First, we incorporate fecal transplants and identify the mechanism by which they treat *C. difficile* infection (CDI). Then, we develop a methodology that endows a microbe with nongeneric attributes within the existing framework; specifically, we add CD sporulation and the developement of antibiotic-resistant strains of CD. By better reflecting the clinically relevant properties of CDI we can “personalize” a mathematical model to a given disease; this construction of generic yet customizable models will be relevant for personalized healthcare models in years to come.

## Introduction

Microbiota are covertly instrumental in bodily functions including immune response [1] and colonization resistance [2, 3]. Some diseases are associated with an imbalanced microbiome, due to disproportionate regulatory action of the host in response to the microbiome composition [4]. Ironically, another pathway to disease is through antibiotic administration, which can dramatically alter microbial composition and diversity, hinder colonization resistance, and subsequently allow for pathogen infection. Specifically in this paper, we focus on antibiotic-induced *C. difficile* infection (CDI), a prevalent nosocomial disease [5, 6].

The advent of high-throughput sequencing provides cheap and accurate time-series abundance data of interacting microbial populations, which can then inform dynamic models that extrapolate system behavior [7, 8]. One idealization of interacting species is the generalized Lotka-Volterra (gLV) model, which assumes that the competitive dynamics of a system are entirely captured through pairwise (inter-species) and self (intra-species) interactions [9]. The gLV model ignores explicit external factors like availability of organic compounds, temperature, or location, but it is the most general possible second order differential equation that describes interacting populations, with some reasonable biological constraints.

Approximating microbiome dynamics as a gLV system is a first step towards quantifying the complex interactions between competing microbes. Inarguably this model misses many subtle, non-competition based, interactions: for example, a non-abundant type of bacteria (e.g. *Escherichia*) may produce proteins vital to general bacterial function (e.g. pili production) [10], but this contribution would not explicitly appear in the model.

In this paper we simulate the prevalence of *C. difficile* (CD) in the microbiome with a generalized Lotka-Volterra model. The work by Stein *et al.* [11] and Buffie *et al.* [12] serves as a point of departure, from which we develop a framework for evaluating the efficacy of different treatment protocols for CDI. This framework develops causal relationships between simulated therapies and microbiome compositions and also explores how bacterial adaptations such as sporulation and antibiotic-resistant mutation may be added to the gLV model. These clinically motivated approaches explain distinct qualitative aspects of CDI that are otherwise unexplored or inconsistent with previous models.

We begin by discussing the clinical background and existing models of CD infection, including the mathematical model we use in this paper, and by describing our in-silico implementations of CD treatments. Then we numerically construct phase diagrams that depict the available behaviors of the simulated system, implement in-silico clinical therapies for CDI, and quantitatively track the efficacies of these therapies. Lastly we describe how to include mechanisms for sporulation and mutation in our model, and evaluate their impacts on the efficacy of antibiotic treatment. Through these techniques, we reveal the importance of timing on the efficacy of fecal microbiota transplantation (FMT) and additionally recover the clinical recommendation for pulsed antibiotic administration when treating CD. Finally, we wield this framework to explain experimental FMT outcomes [13], validate simulated results, and propose future experiments.

The era of personalized medicine and prevalence of high-throughput sequencing will demand accurate microbiome models that can predict, diagnose, and recommend treatment for microbiome disease, and the framework developed in this paper builds upon existing models [14] to progress towards this goal.

### Background

CD is a spore-forming bacterium that can produce toxins which cause CD associated diarrhea, afflicting three million people each year [15]. CDI is especially common in the elderly and in patients who are prescribed antibiotics, since antibiotics deplete the microbiome so that ingested spores of CD— often acquired in healthcare facilities or nursing homes— may invade the vulnerable microbiome [16].

The link between antibiotic treatment, CDI, and microbiome composition was investigated by Buffie *et al.* [12] in a study that gathered mouse time-series phylogenetic data via high-throughput 16S rRNA sequencing. In the study three scenarios were considered, in which the mice were either left alone as a control, exposed to CD, or dosed with the antibiotic clindamycin and subsequently exposed to CD. Each scenario was performed in triplicate and consisted of around 10 time points spanning four weeks, and each time point consisted of thousands of phylogenetic 16S rRNA gene sequences which were mapped to taxonomic species and tallied. The study found that after antibiotic administration of clindamycin the mouse microbiome was less diverse (in terms of the Shannon diversity index) and vulnerable to CDI, which is consistent with clinical observations of humans who develop CDI [15, 16]. Because the anatomies of mice and humans are similar [17] and the microbiomes of both species react to changes in diet in a similar manner [18], it is common to treat the mouse model as a proxy for human CDI.

In a first attempt to model the relationship between CDI and antibiotic treatment, Stein *et al.* [11] proposed a generalized Lotka-Volterra (gLV) model to explain the interactions between different microbes. The parameters for this model were fit with the previously mentioned data from Buffie *et al.* [12]. To reduce dimensionality, Stein *et al.* assumed that bacteria within a given genus behave similarly, and consolidated the species-level data into genus-level data. The parameter fitting procedure was tested on in-silico data, and the fitted parameters satisfied biologically reasonable restrictions. This model— described in more detail in the text surrounding Eq (1)— produces microbiome composition trajectories which allow for simulated antibiotic treatment or exposure to CD. The Spearman rank correlation, a measure comparing the predicted microbe abundances with the experimentally measured abundances, was 0.62 (the largest achievable value is 1), and simulated trajectories for each microbe typically matched experimental trajectories within an order of magnitude. Especially, the model preserved the clinical and experimental conclusion that microbiomes treated with the antibiotic clindamycin were vulnerable to CDI.

In this paper, we start from a gLV model with previously fitted parameters [11], analyze the steady states, and then build upon this model to explore clinically motivated adaptations. In particular, we focus on simulated remedial treatments that can avoid or reverse *C. difficile* infected steady states, which we interpret as microbiomes suffering CDI.

## Models and methods

### Generalized Lotka-Volterra equations

The generalized Lotka-Volterra equations track the abundance of *N* populations *x*_*i*_ through time; in our case, the populations are *N* − 1 genera plus the bacterial species CD. They read, for *i* ∈ 1, …, *N*,

$$\begin{array}{c} \frac{d}{dt}x_{i}\left(t\right)=x_{i}\left(t\right)(\mu_{i}+\sum_{j=1}^{N}M_{ij}x_{j}\left(t\right)+\varepsilon_{i}u\left(t\right))\;. \end{array}$$ (1)

The dynamics of each population are of the same form, so the distinct individual trajectories are entirely determined by the choices of parameters and initial conditions. The parameters and initial conditions that are used to generate each figure are given in Table A of S1 Appendix. For a population *x*_*i*_, *μ*_*i*_ describes that population’s self-growth while *M*_*ij*_ describes the pairwise effect of population *j* on population *i*, an interaction that can be interpreted as mutualistic, commensalistic, or parasitic. Lastly, *ε*_*i*_*u*(*t*) is an external forcing term, which in our model represents the effect of an administered antibiotic *u*(*t*) operating with efficacy *ε*_*i*_. In all, Eq (1) accounts for zeroth, first, and second-order terms, and approximates the competitive dynamics as a power series of the individual populations.

**Fig 1:**
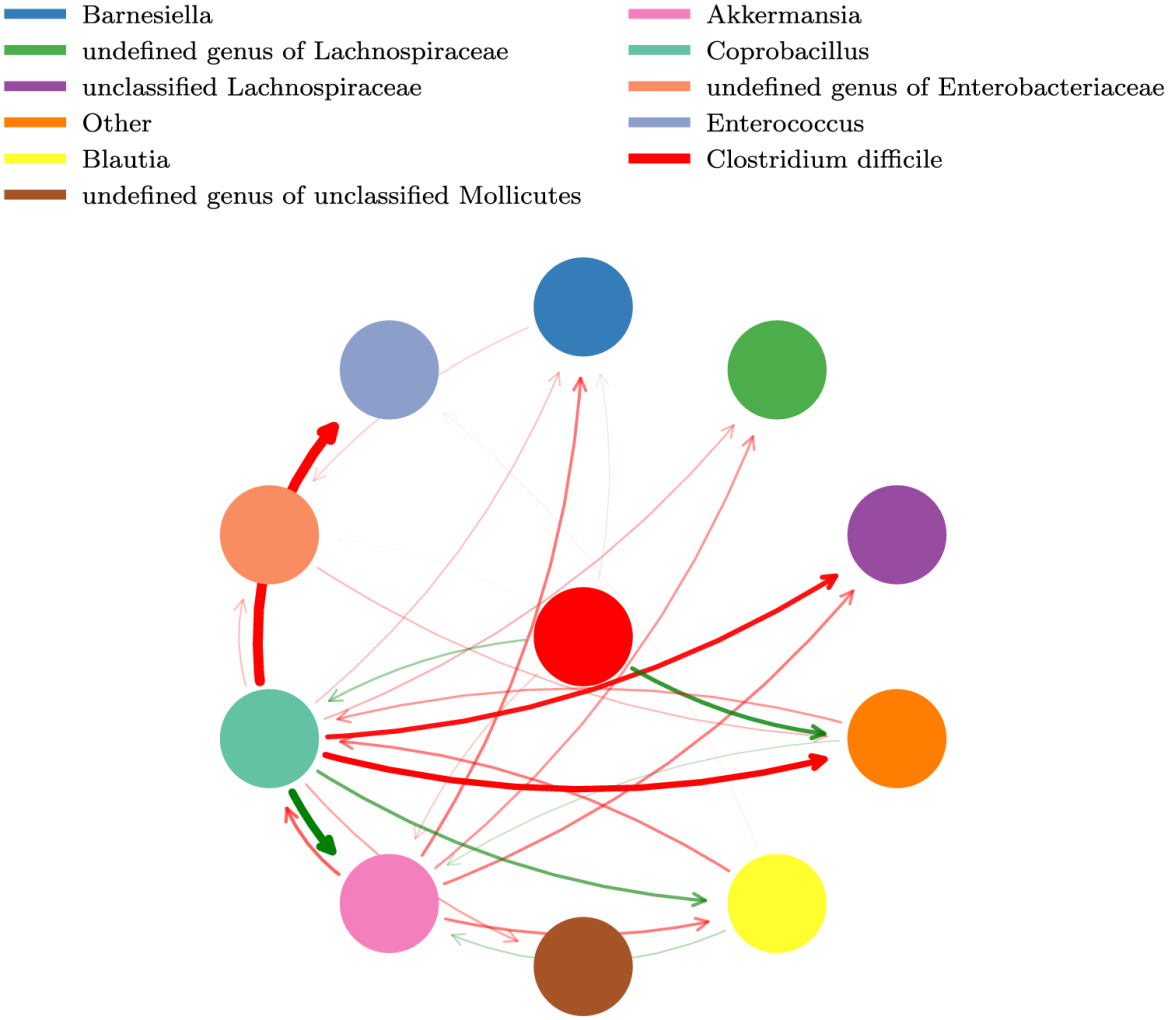
Pairwise interactions between bacterial populations may be interpreted as a microbial food web. An arrow from population *j* to population *i* represents the effect of *j* on the growth of *i*, which we equate to the interaction term *M*_*ij*_ in a generalized Lotka-Volterra model, Eq (1). The width and opacity of an arrow are proportional to |*M*_*ij*_|, and positive interactions (*M*_*ij*_ > 0) are green while inhibitory interactions (*M*_*ij*_ < 0) are red. *M*_*ij*_ was fit in [11] using experimental mouse data from [12]. To reduce dimensionality, bacterial species of the same genus are consolidated into one population; the exception is *C. difficile* (CD), which is a single bacterial species. CD, the culprit behind *C. difficile* infection (CDI), is colored red and located in the center of the food web.

The procedure for parameter fitting is explained in detail and performed by Stein *et al.* [11]. Briefly, the fitted parameter values satisfy *μ*_*i*_ > 0 and *M*_*ii*_ < 0 for each *i*, so that in isolation each population will grow and eventually self-limit. Most but not all microbial groups are inhibited by the antibiotic clindamycin. Since the interactions between populations have no clear hierarchy, we interpret the gLV model as microbes on the same trophic level competing for a shared resource— the pairwise interactions, then, effectively describe a food web which we visualize in Fig 1. While dynamical systems such as this one may in principle display an array of behaviors, with these fitted parameters we have only observed trajectories that approach biologically reasonable steady states (e.g. no periodic orbits have been observed); if we interpret the negative values in *M*_*ij*_ as negative covariances between populations, then this stability is consistent with the covariance effect [19].

### Simulation of CDI treatments

In clinical practice, CDI is defined by the presence of toxigenic CD or of CD toxins in a patient experiencing diarrhea— since there are asymptomatic carriers of CD the mere presence of CD is not sufficient for diagnosis [16]. However, since the model Eq (1) does not predict toxigenicity or toxin production, for the purposes of this paper we equate CDI to the prolonged presence of CD in a simulated microbiome.

Stein *et al.* [11] investigated the existence and stability of steady states for the system Eq (1). Additionally, they found that for some initial compositions, antibiotic administration can alter a microbial composition to the degree that the composition becomes susceptible to CD colonization. Building upon their work, we propose the following three clinically relevant interventions and their corresponding in-silico implementations:

1. **inoculation** with CD at time *t*_*I*_, corresponding to **x**(*t*_*I*_) ↦ **x**(*t*_*I*_) + **x**_*c*_ where **x**_*c*_ is purely composed of CD,
2. **antibiotic administration**, corresponding to a *u*(*t*) that is (unless otherwise specified) a unit pulse of concentration *c* at *t* = 0, and
3. **transplantation** of CD-resilient microbiota into a CD-susceptible microbiome at time *t*_*T*_, corresponding to **x**(*t*_*T*_) ↦ **x**(*t*_*T*_) + **x**_*IC*_, where **x**_*IC*_ is a transplant composed of a CD-resilient initial condition.

We refer to a simulation which implements any combination of these external interventions as a *treatment scenario*.

Simulations are run in Python with the scipy package and the scipy.integrate.odeint function, which uses ordinary differential equation solver lsoda from odepack, written in FORTRAN. This solver adaptively switches between stiff and non-stiff solvers, and simulations are run with an absolute tolerance of 10^−12^. The code used to generate the figures in this paper is freely available at https://github.com/erijones/simulated_CDI_with_gLV.

### Simulated microbial transplants

Clinically, an external microbial transplant seeks to rejuvenate an unhealthy microbiome by infusing “healthy” microbes into the unhealthy patient. The infused samples typically consist of probiotics or a microbiome (often fecal) sample from a healthy subject [20]. Microbial transplants can confer attributes (e.g. obesity) from the donor to the donee [21], so in some sense a microbiome transplant is seeking to confer *CD-colonization resistance* from a CD-resilient donor to a CD-susceptible donee. Since antibiotics tend to be ineffective in treating CDI and additionally can facilitate the growth of drug-resistant mutant strains of CD by providing them with a selective advantage, fecal transplants are becoming an increasingly popular CDI treatment [15].

In our implementation we simulate transplants made of CD-resilient initial conditions, and demonstrate how these treatments can guide the system into a desired (i.e. noninfective) steady state. We model the administration of a transplant of some external microbial source **v** at time t* as

$$\begin{array}{c} \frac{dx(t)}{dt}=f\left(x\right)+v\;\delta \left(t-t^{*}\right)\;, \end{array}$$ (2)

where **f**(**x**) entirely encapsulates the right-hand sides of the gLV equations of Eq (1) in vector form and *δ*(*t*) is the Dirac delta function, which will serve to instantaneously add the transplant **v** to the microbial community **x** at time t*.

### Sporulation

Under environmental pressures CD can sporulate, entering a defensive state of dormant spores that maintain the genetic information of CD while functioning at a fraction of the vegetative cell’s metabolism. These spores are resilient to antibiotics, and CD sporulation may be induced by environmental stressors such as heat [22] and alcohol [16]. While the entire gamut of environmental conditions that induce sporulation is not yet known [23], there is some evidence that in murine models antibiotics may induce sporulation [15]. The toxin-producing types of CD prevalent in nosocomial infections are notoriously difficult to kill, and their resilience has in part been attributed to sporulation [15].

Mathematically, sporulation can be modeled by creating a population of spores that, through conversion of active CD, grows when environmental conditions are harsh and declines when conditions are mild. This implementation is inspired by the treatment of latently infected T-cells in SIR models of HIV, in which the latently infected T-cells effectively hide from the immune response in the same way that the inert spore cells are uneffected by the presence of antibiotics and other microbes [24]. To capture sporulation, we augment the basic model Eq (1) by introducing a spore compartment *s*(*t*) so that the populations of the original gLV model become

$$\begin{array}{c} \begin{array}{cc} \frac{\text{d}}{\text{d}t}x_{i}\left(t\right) & =x_{i}\left(t\right)(\mu_{i}+\;\sum_{j}M_{ij}x_{j}\left(t\right)+\varepsilon_{i}\;u\left(t\right))\;,\\ \frac{\text{d}}{\text{d}t}x_{c}\left(t\right) & =x_{c}\left(t\right)(\mu_{c}+\;\sum_{j}M_{cj}x_{j}\left(t\right)+\varepsilon_{c}\;u\left(t\right))+\beta s\left(t\right)\left[u\left(t\right)<u_{spor}\right]\;,\;\text{and}\\ \frac{\text{d}}{\text{d}t}s\left(t\right) & =\alpha \;x_{c}\left(t\right)\left[u\left(t\right)\geq u_{spor}\right]-\beta s\left(t\right)\left[u\left(t\right)<u_{spor}\right], \end{array} \end{array}$$ (3)

where the terms in square brackets should be interpreted as conditional statements that return 1 if true and 0 if false.

In Eq (3), we assume that the background microbes (which we define as the bacteria that are not CD) are uneffected by the presence of the inert spores. In the presence of antibiotics bacterial growth often acts as a step function, growing or not growing if the antibiotic concentration is lower or higher than the bacteria’s minimum inhibitory concentration (MIC) [25]. We similarly model the inflow and outflow of spores as a step function, where sporulation or germination occurs if the antibiotic concentration is larger or smaller than some threshold *u*_*spor*_. Since the spores are robust, we assume they have no death rate. We assume that some proportion *α* of the CD normally killed by antibiotics are converted to spores, so there is no explicit *α* term in the CD growth term, and as a consequence of this we require *α* < *ε*_*c*_*u*(*t*). The experimental methods used to measure CD sporulation are not yet standardized, so there is no clear consensus on the rate of CD sporulation [22]; therefore, the sporulation parameters *α*, *β*, and *u*_*spor*_ must be considered in a qualitative fashion.

### Mutation

The final augmentation we add to the gLV model is antibiotic-resistant mutation, which is culpable for many of the difficulties in treating CDI [26]. Existing antibiotic resistance models for both within-host [27] and between-host [28] versions of antibiotic-resistance typically only consider isolated bacterial systems which include only the native and mutant strains of a single bacterial species. Since we consider mutation in the gLV framework, in this paper we are able to probe the more realistic scenario of mutation occurring within a complex microbial community.

We modify the standard gLV model in Eq (1) to include terms that allow for mutation of CD into an antibiotic-resistant mutant strain of CD, denoted *x*_*m*_(*t*), so that the microbial dynamics are described by

$$\begin{array}{c} \begin{array}{cc} \frac{\text{d}}{\text{d}t}x_{i}\left(t\right) & =x_{i}\left(t\right)(\mu_{i}+\;\sum_{j}M_{ij}x_{j}\left(t\right)+\varepsilon_{i}u\left(t\right))\;,\\ \frac{\text{d}}{\text{d}t}x_{c}\left(t\right) & =x_{c}\left(t\right)(\mu_{c}+\sum_{j}M_{cj}x_{j}\left(t\right)+\varepsilon_{c}u\left(t\right))-k\;x_{c}\left(t\right)\;,\;\text{and}\\ \frac{\text{d}}{\text{d}t}x_{m}\left(t\right) & =x_{m}\left(t\right)(\mu_{m}+\sum_{j}M_{mj}x_{j}\left(t\right))+k\;x_{c}\left(t\right). \end{array} \end{array}$$ (4)

In addition to the standard gLV pairwise interactions, the background microbes *x*_*i*_ of Eq (4) now interact with the CD mutant *x*_*m*_ via the *M*_*im*_ term. Following existing mutation models [28], we (1) group all potential antibiotic-resistant mutations into the one mutant population *x*_*m*_ and (2) neglect the possibility of mutation from a mutant strain *x*_*m*_ back to the native strain *x*_*c*_. Furthermore, we assume that the mutations are fully resistant to antibiotics and so we omit the *ε*_*m*_ term in Eq (4). While other candidate models for antibiotic-resistant mutation exist and have been examined [29], here we focus on embedding this particular implementation of single-strain mutation into the gLV framework; other types of mutation models may be implemented in a similar way.

Since we are extrapolating beyond the mouse data collected in [12], it is not surprising that the mouse microbiome data does not distinguish between native and mutant strains of CD. Antibiotic resistant strains of CD are already rampant: one survey found that close to half of tested CD strains were resistant to at least one antibiotic, and about one quarter of tested strains were resistant to multiple antibiotics [30]. However, since the antibiotic susceptibility of CD *ε*_*c*_ is non-zero, we assume that the administered CD used to inoculate the mice is antibiotic-sensitive.

## Results

### Mapping system behaviors

**Fig 2:**
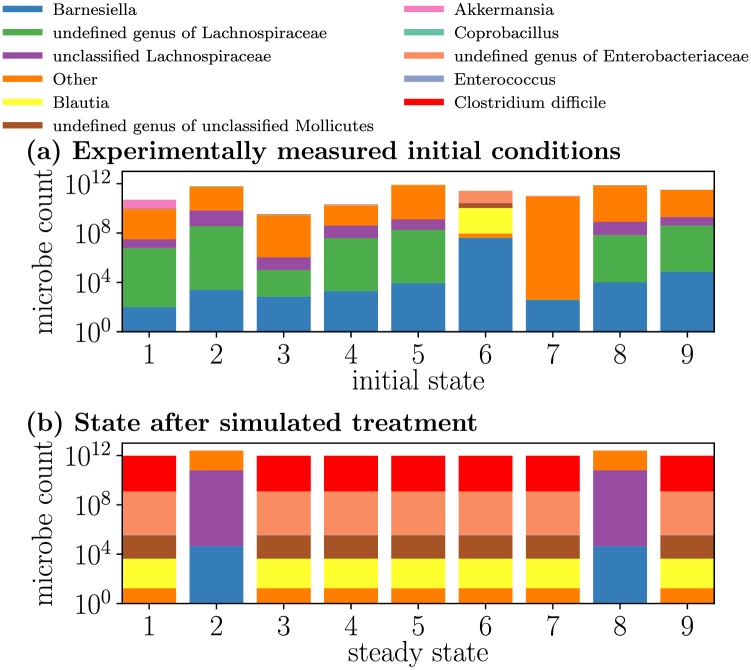
Diverse initial conditions respond similarly for a simulated treatment. Microbiome compositions are simulated forward in time from experimentally determined and diverse initial conditions (a), but all initial conditions eventually equilibrate to only two steady states (b) for this particular treatment scenario. Initial conditions are experimentally measured microbiome compositions from mice [12] and are time evolved according to the generalized Lotka-Volterra model, Eq (1), with parameters fit in [11]. In this simulated scenario, the system is administered 1 dose of antibiotic on day 0 and inoculated with the infectious CD (colored red) on day 10.

We first demonstrate the available behaviors of the system described by Eq (1). In Fig 2 we evolve our system from the nine distinct initial conditions experimentally measured by Stein *et al.* [11] for one particular treatment scenario, in which all initial conditions are initially treated with antibiotics and later inoculated with CD. All but one of these initial conditions are free of CD, and the remaining initial condition (IC 8) has a trace amount of CD. Despite the diverse composition of the initial conditions, under this treatment scenario the simulated trajectories evolve into only two steady states.

**Fig 3:**
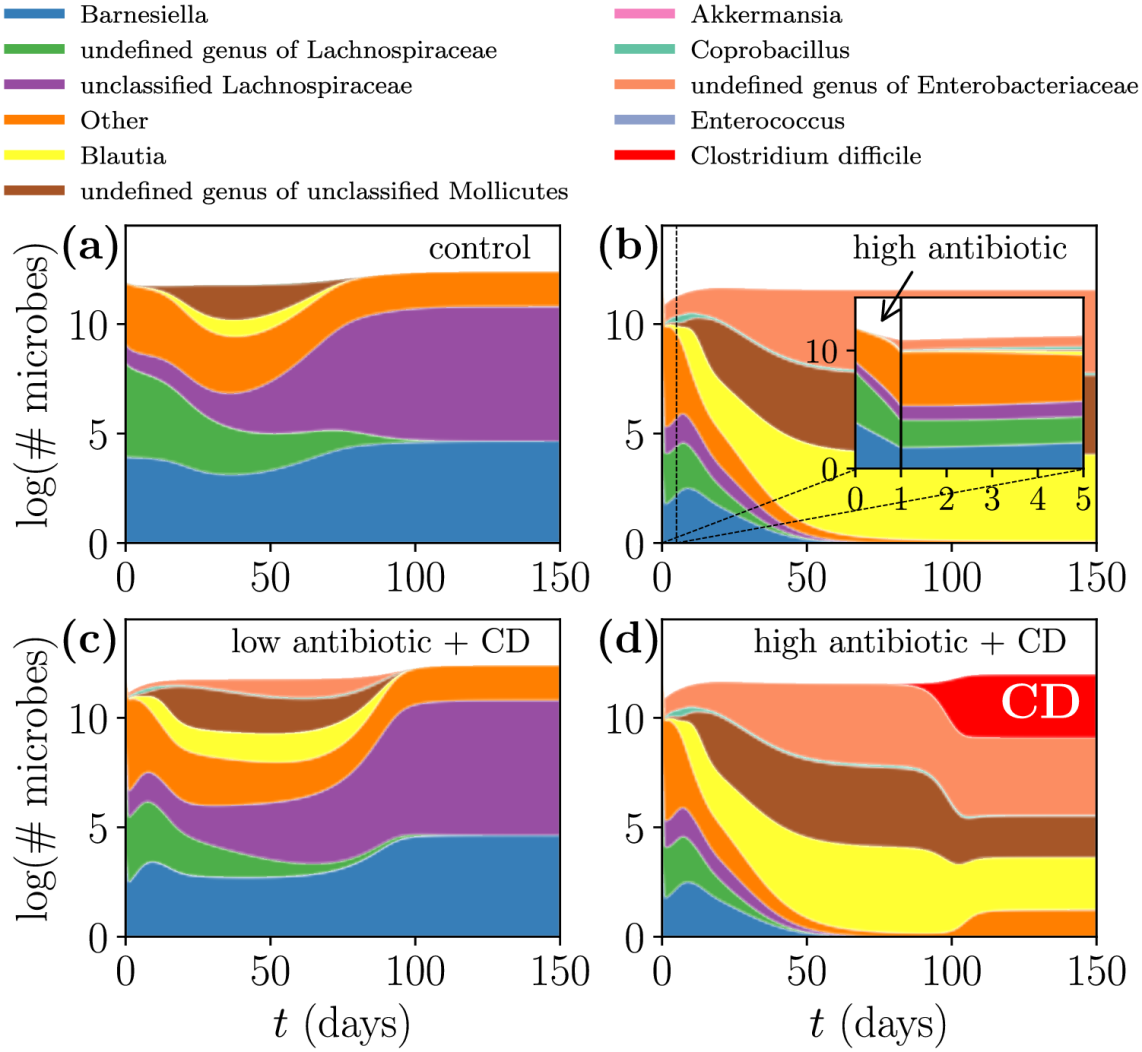
External intervention can alter the steady state a given initial condition achieves. All panels originate from the same initial condition, but different panels correspond to different interventions: (a) no interventions occur; (b) one dose of antibiotic is administered at day 0 (inset: the microbial dynamics during the first 5 days, in response to the one-day administration of antibiotics); (c) half of a dose of antibiotic is administered at day 0, and then at day 10 the system is inoculated with CD; (d) one dose of antibiotic is administered at day 0, and then at day 10 the system is inoculated with CD. The growth of *C. difficile* (colored red) is encouraged by antibiotic treatment, since the antibiotics deplete the other microbes to a level at which *C. difficile* gains a foothold.

Then, in Fig 3 we apply four different treatment scenarios to one initial condition and identify three different reachable steady states, indicating that the initial conditions can be sensitive to which treatment scenario is applied. In this paper, within a single simulation microbe counts can vary by more than two orders of magnitude. For clarity, in our figures we plot the total microbe count on a log scale (where the total microbe count is the sum of all of the microbes in each microbial population), and then at each time we linearly color each microbial population according to its proportion at that time, so that at a given time regions of equal size correspond to equal microbe counts. The treatment scenarios that result in Fig 3 mirror the experimental mouse treatments [12] and include a control, high dosing with antibiotic (the inset of Fig 3b depicts the initial microbial response to antibiotics), low dosing with antibiotic followed by inoculation with CD, and high dosing with antibiotic followed by inoculation with CD. While the log scaling disguises changes in total microbe count between the different steady states, the steady state of Fig 3a contains seven times as many microbes as the depleted (in microbe count) steady state of Fig 3b, and contains more than twice as many microbes as the infected steady state of Fig 3d (for details on steady state compositions refer to Table B of S1 Appendix). This figure elucidates the mechanism for CDI: antibiotic-induced microbiome depletion followed by opportunistic CD colonization.

Taken together the complementary results of Figs 2 and 3 indicate that (1) for a given treatment scenario there are a limited number of achievable steady states across all initial conditions, and (2) for a given initial condition there are a variety of steady states that may be achieved across different treatment scenarios. Since the model was fit with data collected over a 30 day period but the obtained steady states are often slow to equilibrate (e.g. around 100 days in Fig 3), we should proceed with caution when extrapolating the model [31]. However, since the collected experimental data [12] roughly equilibrates by day 30, and because experimental validation on longer time scales is difficult to obtain, we follow convention [32] and study long-term system behavior through steady state analyses.

In the four weeks before the mouse experiment the mice were identically housed and fed, and during the experiment the microbial compositions of mice in the control group were approximately constant over time [12]. Hence, what we consider “initial conditions” may also be interpreted as steady states compositions of the mice before any external intervention. However, the gLV model Eq (1) does not capture these initial conditions as steady states. Over the course of the 13-day control group experiment the measured bacterial abundances maintained a relatively stable composition, with the 7 or 8 colonized bacteria varying by less than an order of magnitude over the course of the experiment. However, the model Eq (1) predicts that the same control group initial conditions (ICs 2, 5, and 8) will tend towards a simpler steady state that consists of only 3 bacteria.

This inconsistency demonstrates two limitations of the gLV model: the paucity of steady states, and the likelihood of their stability. For a generalized Lotka-Volterra system of *N* species there are 2^*N*^ steady states, each corresponding to a different subset of bacteria— hence, there is just one steady state that consists exclusively of the 7 overlapping bacteria of the control group. Since there is variation between the control experiments, there can be no steady state that would simultaneously and precisely fit all three control trials. Furthermore, even if this steady state were relatively accurate for each trial it is unlikely that it would be stable: Stein *et al.* [11] found that 98% of the steady states of this system were unstable. Despite the fact that unperturbed initial conditions are not stable steady states, other qualitative features of the model (including antibiotic-induced depletion of the microbiome and opportunistic CDI) indicate the model’s utility in modeling CDI.

**Fig 4:**
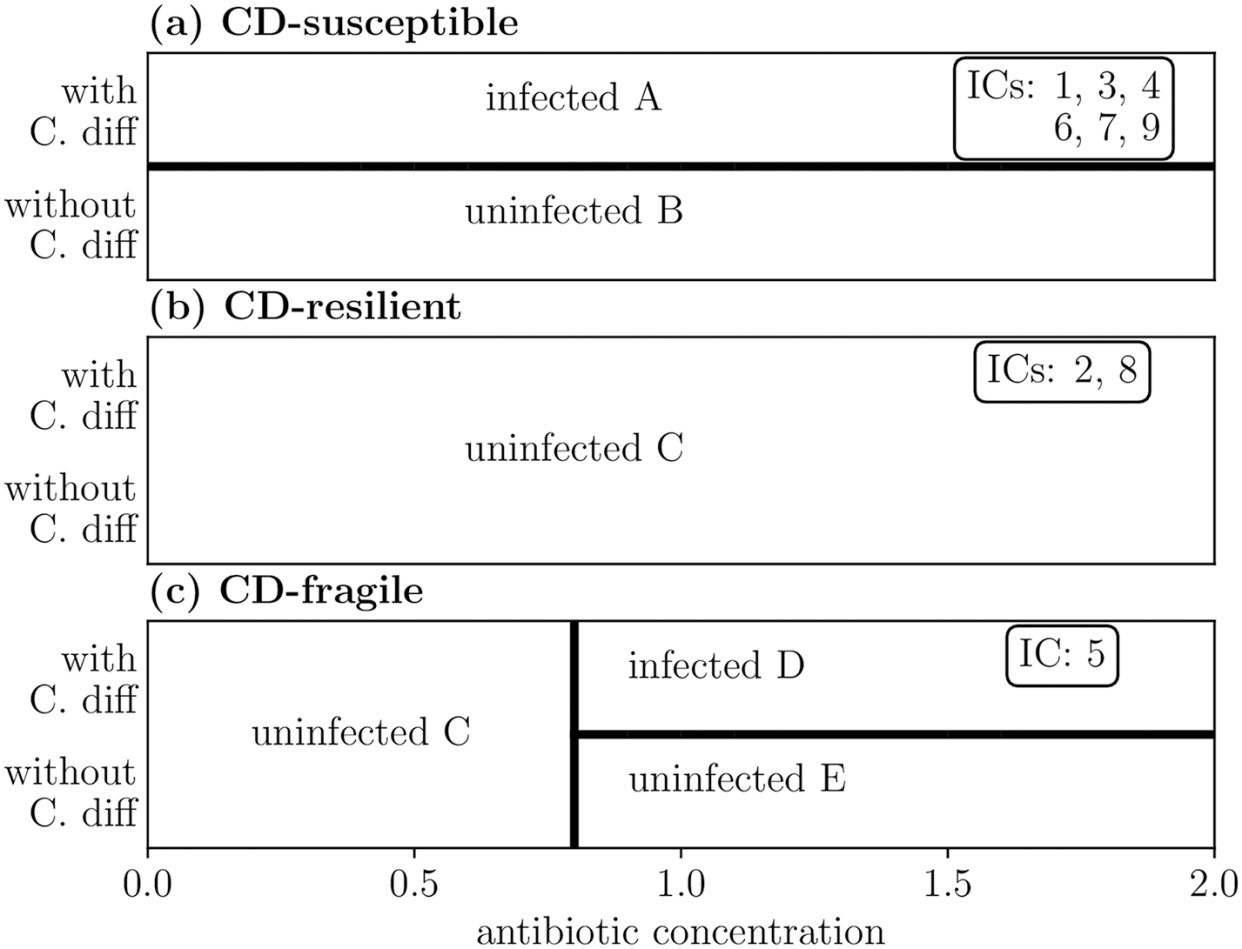
Phase diagram of reachable steady states from initial conditions. (a) Six initial conditions (ICs) are susceptible to *C. difficile* (*CD-susceptible*), resulting in infected steady state A if inoculated with any amount of CD. (b) Two initial conditions are *CD-resilient* and always remain in uninfected steady state C regardless of CD exposure. (c) One initial condition displays more complex behavior, becoming susceptible to CD only after being treated with a sufficient dose of antibiotics (*CD-fragile*). The steady states of the four external interventions of Fig 3 correspond to different regions of the CD-fragile phase diagram (c). The phase boundaries of Fig 4 are robust to the amount of CD inoculum (ranging from 10^−10^ to 1 in nondimensionalized units) as well as to the timing of CD inoculation (ranging from on day 1 to on day 100). For details, refer to Table A of S1 Appendix.

To summarize the available system dynamics, we construct the phase diagrams in Fig 4 by systematically sweeping through treatment scenarios for each initial condition; specifically, we vary the concentration of antibiotic treatment and whether the system is exposed to a small amount of CD.

Though we simulate nine initial conditions (ICs), the phase diagrams for some initial conditions are redundant. We classify the phase diagrams of Fig 4 as (a) *CD-susceptible*, ICs which become infected upon exposure by CD regardless of antibiotic usage; (b) *CD-resilient*, ICs which are not infected by CD regardless of antibiotic usage; and (c) *CD-fragile*, ICs which switch from CD-resilient to CD-susceptible upon sufficient administration of antibiotic (an antibiotic concentration of approximately 0.71). We label the five reachable steady states A through E, categorize them as CD-infected or CD-uninfected, and plot their compositions in S1 Fig. Each phase diagram is composed of a number of treatment scenarios; for each treatment scenario, a 1-day pulse of antibiotic with varying antibiotic concentration is administered on day 0, and then a small amount of CD may be administered on day 10 depending on whether the scenario is with or without CD. For reference, the experimental antibiotic dose was normalized in [11] to a 1-day pulse of antibiotic concentration 1.

With the phase diagrams of Fig 4, we may now identify the initial condition plotted in Fig 3 as CD-fragile. Furthermore, the steady states of Fig 3a–3d correspond, respectively, to steady states C, E, C, and D of Fig 4. Notably, IC 8 is CD-resilient despite the fact that the initial condition contains a small amount of CD; in fact, according to the fitted interactions the presence of CD promotes the growth of microbes that inhabit the uninfected steady state. Therefore, the isolated presence of CD inhibits colonization of an infected steady state.

One key takeaway from this survey of model behaviors is that there is no *a priori* obvious predictor for whether an initial condition will be CD-susceptible, CD-resilient, or CD-fragile, even with knowledge about the microbial food web. Often, the complex interplay of microbial interactions can lead to unexpected and even counterintuitive results.

### Invadability of CD

In the numerical phase diagrams of Fig 4 we observe different regimes for different initial conditions, but we can substantiate this phenomenon analytically as well. We label steady state A in Fig 4 by $x_{A}^{*}$, and similarly label all other steady states. After all antibiotic has been administered, we perform a perturbative analysis of the uninfected steady states by introducing a small amount of CD (notated *x*_*c*_(*t*)) to the uninfected steady state x*. This CD will invade the steady state only if $[\frac{d}{dt}x_{c}\left(t\right)]{|}_{x^{*}}>0$. Since the introduced *x*_*c*_(*t*) is positive, we may discern the invadability of an uninfected steady state x* by the sign of *I*(x*), defined to be

$$\begin{array}{c} I\left(x^{*}\right)\equiv \frac{1}{x_{c}\left(t\right)}\left[\frac{\text{d}}{\text{d}t}x_{c}\left(t\right)\right]{|}_{x^{*}}={\left(\mu +Mx^{*}\left(t\right)\right)}_{c}. \end{array}$$ (5)

Here, we have rearranged Eq (1), removed the antibiotic dependence *u*(*t*), consolidated all the *μ*_*i*_ and *M*_*ij*_ into their respective vector and matrix forms ***μ*** and *M*, and consolidated the individual populations *x*_*i*_(*t*) into their vector form **x(t)**. Notationally, the subscript *c* denotes the value of a vector corresponding to the index of CD. While magnitude of the invadability |*I*(x*)| will correspond to the initial rate at which CD will grow or decay, only the sign of *I*(x*) is relevant in determining long-term susceptibility or resilience to CD.

In Table 1 we compute and compile this invadability for each of the three uninfected steady states $x_{B}^{*}$, $x_{C}^{*}$, and $x_{E}^{*}$. This table also provides the size of each steady state, where size is interpreted as the sum of all the bacterial populations (here written as the 1-norm). These conclusions provide analytic justification for why some initial conditions are susceptible to CD while others are not, and complement the phase diagrams in Fig 4.

**Table 1:** Analytic justification for CD-susceptibility. The ability of CD to invade the three uninfected steady states $x_{B}^{*}$, $x_{C}^{*}$, and $x_{E}^{*}$ depends upon the sign of $I\left(x^{*}\right)\equiv \frac{1}{x_{c}\left(t\right)}{\left[\frac{\text{d}}{\text{d}t}x_{c}\left(t\right)\right]}_{x^{*}}$: a positive value indicates a CD-susceptiblity, while a negative value indicates CD-resilience. This result follows from analysis of Eq (1).

steady state	interpretation (*I*(x*))	‖x*(*t*)‖_1_
$x_{B}^{*}$	CD-susceptible (0.24)	3.238
$x_{C}^{*}$	CD-resilient (-0.86)	24.770
$x_{E}^{*}$	CD-susceptible (0.28)	3.546

CD is predominantly inhibited by the existence of other microbes (mostly, *M*_*cj*_ < 0) and so a larger |x*(*t*)| will tend to inhibit the growth of CD. Additionally, microbes tend to be inhibited by antibiotics (mostly, *ε*_*i*_ < 0). Together, these tendencies allude to a mechanism of CDI whereby antibiotic administration depletes the microbiome and induces CD susceptibility.

While Table 1 indicates that the reachable CD-susceptible steady states are smaller than CD-resilient steady states, the size of the initial condition had little effect on the overall steady state profile: growing or shrinking the initial condition sizes only marginally modified the resulting phase diagrams. Hence, the different steady states are robust to variations in initial condition size.

Having exhaustively explored the basic behaviorial regimes of Eq (1), we now implement in-silico two commonly administered real-world medical interventions: *fecal microbiome transplantation* and *antibiotic administration*.

### Simulated microbial transplants

**Fig 5:**
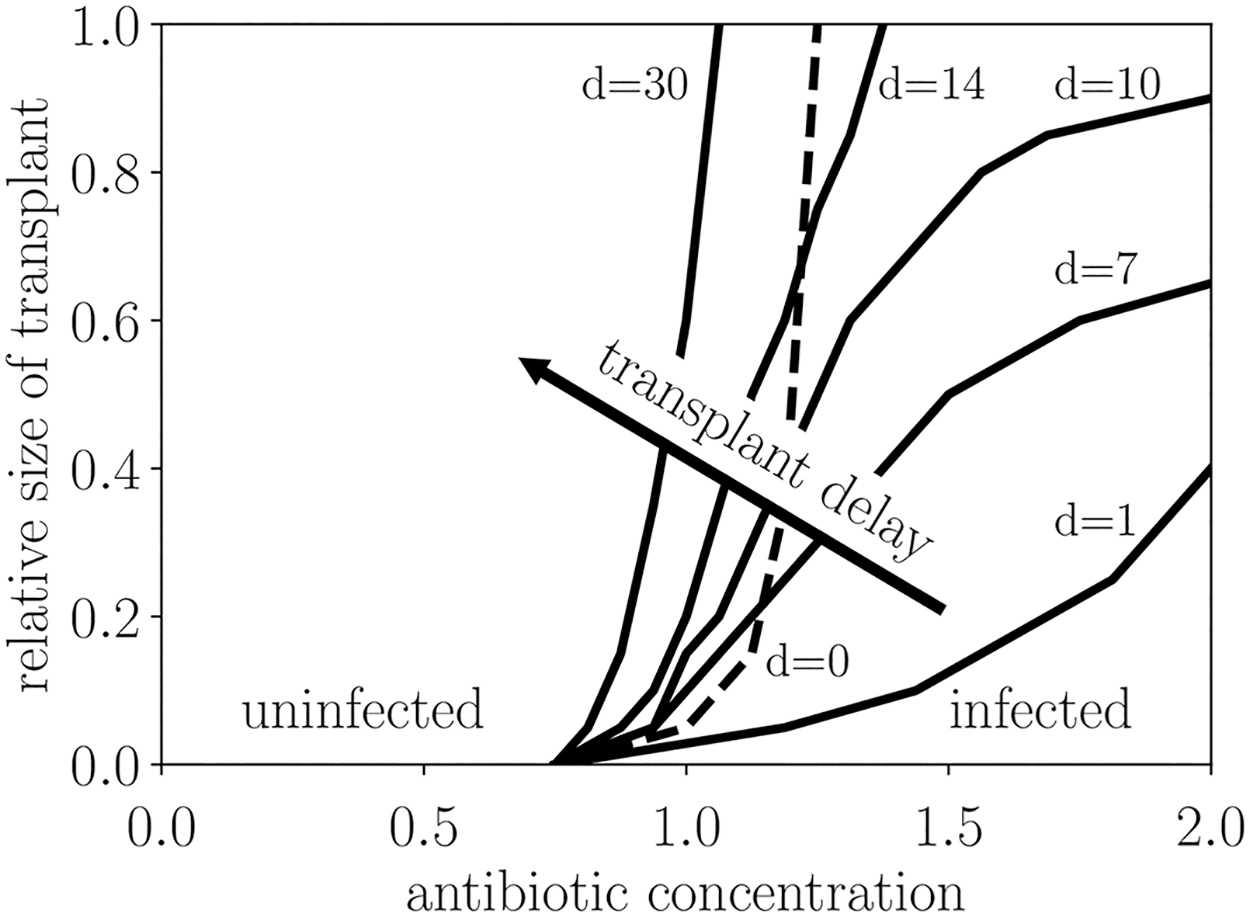
Administration of microbial transplants can ward off infected steady states. Starting from the CD-fragile initial condition, antibiotics of varying antibiotic concentration are administered on day 0, and the system is exposed to CD on day 1. Then, a “healthy” transplant made up of the CD-resilient initial condition 8 is infused on day *d*. The *infected* region corresponds to infected steady state D, and the *uninfected* region corresponds to uninfected steady state E. Note that a transplant on day 0 (dashed line), concurrent with the start of antibiotic administration, is less effective than a transplant on day 1. A relative transplant size of 1 corresponds to a transplant that has the same size as the initial condition that the transplant was derived from. The phase boundaries of Fig 5 are robust for small amounts of CD inoculum (ranging from 10^−10^ to 10^−8^ in nondimensionalized units), but for larger amounts of CD inoculum (ranging from 10^−5^ to 10^−2^) transplants become more effective at all timings, requiring a smaller transplant to overcome a larger antibiotic dose. For details, refer to Table A of S1 Appendix.

Following Eq (2), we choose a microbial transplant **v** that is derived from a CD-resilient donor so that **v** is proportional to the composition of a CD-resilient initial condition, and we choose the donee microbiome to be the CD-fragile initial condition so that the effects of the transplant are more apparent. In the simulation we choose the timing of the treatment scenario to match the clinical counterpart of CDI, in which CD attempts to colonize a microbiome that has been recently depleted by antibiotics: we administer antibiotics on day 0, inoculate with CD on day 1, and insert a transplant on day *d*. By categorizing the resultant steady state as CD-infected or CD-uninfected and sweeping over antibiotic concentrations, relative transplant sizes, and transplant times, we realize the phase diagram in Fig 5.

Fig 5 demonstrates how a transplant can alter the steady state behavior of a system exposed to CD. We can bias the initial condition towards a CD-uninfected steady state with a proper fecal transplant via the mechanism of *steady state conversion*, wherein a transplant can convert a state from CD-susceptible to CD-resilient. This result, consistent with clinical practice, supplies a numerical framing for microbial transplants, narrowing the gap between real-world practice and simulation.

For transplants that are applied after antibiotic administration, this figure indicates that shorter transplant delays lead to more effective transplants. However, a transplant applied concurrently with antibiotic administration on day 0 (labeled d = 0 in Fig 5) is less effective than a transplant applied after antibiotics on day 1. This reflects that antibiotic administration depletes *all* microbes, so a transplant on day 1 will be unsullied by antibiotics whereas applying a transplant on day 0 will lead to the depletion of the aggregate composition.

**Fig 6:**
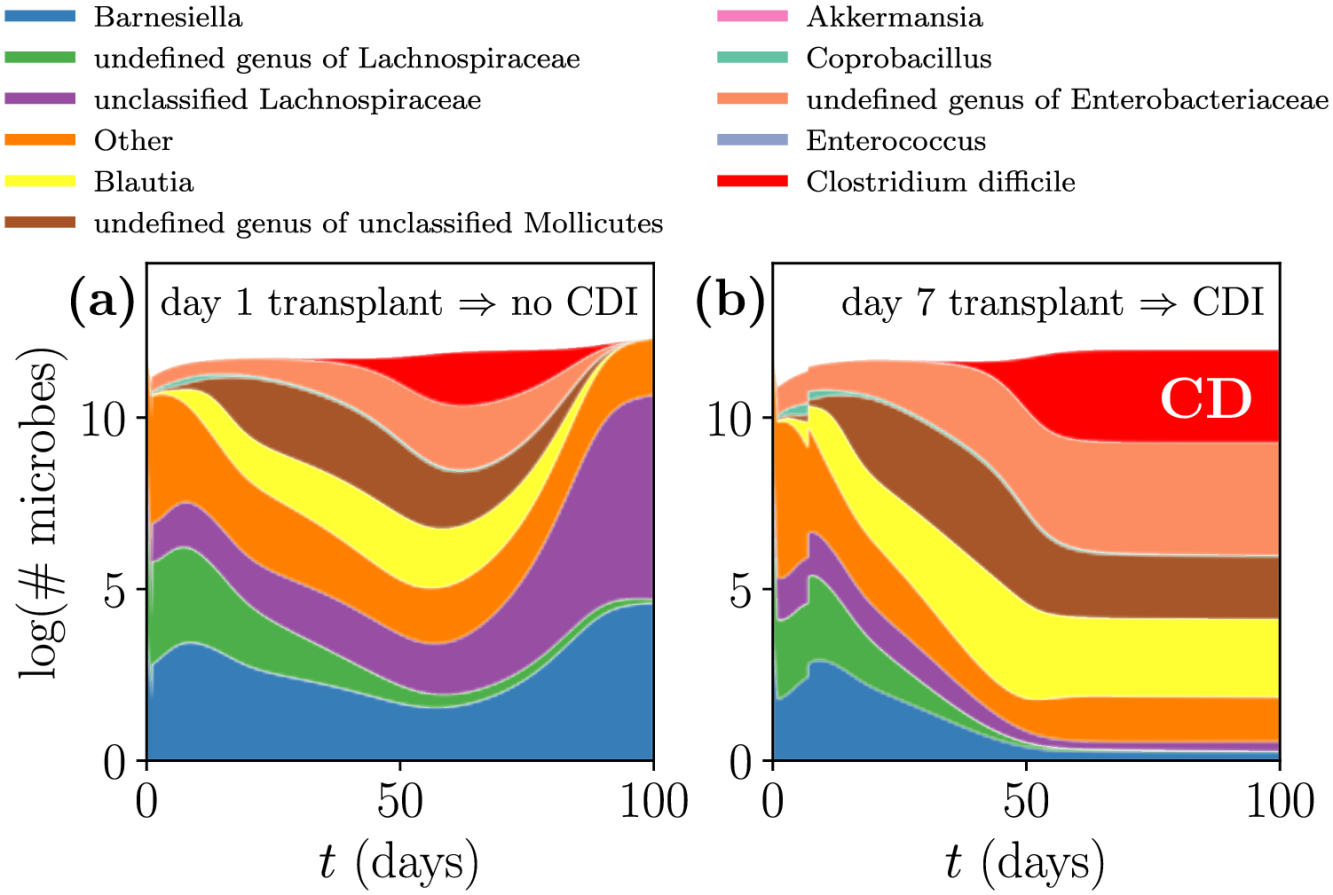
Mechanism of *steady state conversion*. Microbiome compositions are identically dosed with antibiotic on day 0 and inoculated with CD (colored red) on day 1. Transplants of the same size are administered on (a) day 1, leading to a CD-uninfected steady state, and (b) day 7, leading to a CD-infected steady state. Following the antibiotic-induced microbiome depletion, the transplant serves to replenish the microbes responsible for the CD-uninfected steady state (e.g. unclassified Lachnospiraceae, colored purple) while curbing the growth of those responsible for the CD-infected steady state (e.g. Blautia, colored yellow).

In Fig 6 we examine the effect of transplant timing for a fixed antibiotic concentration and transplant size. Steady state conversion is most effective immediately after antibiotic administration, when the depleted microbiome has room to grow. During this time the malleable microbiome is especially responsive to transplants, and introduction of the right collection of microbes can direct the microbiome towards an infection-free steady state. However, as indicated in Fig 3, without any transplant the CD-fragile IC will naturally evolve towards a CD-susceptible steady state: hence, the timing of the transplant is critical, with more immediate transplants being more effective.

We found that out of the measured ICs, the collection of microbes that best deter CDI are derived from IC 8. This transplant replenishes the unclassified Lachnospiraceae (colored purple), which promote constituents of the uninfected steady state while inhibiting Blautia (colored yellow), a key member of the infected steady state. More surprisingly, the existence of CD in IC 8 amplifies the effect of the transplant— the same transplant but without CD was a functional but substantially less effective treatment, and similarly mediocre results were obtained with a transplant derived from the other CD-resilient initial condition (IC 2) as displayed in S2 Fig. This result is due to the deleterious and contradictory effect of CD on the CD-infected steady state. As an aside, note that since IC 8 contains CD, the transplant on day 0 effectively inoculates the system with CD on day 0 rather than on day 1.

While appropriately derived and implemented transplants are effective at reversing CDI, if we had mistakenly used a CD-susceptible donor instead, simulation confirms the intuitive expectation that these results would be flipped. Since these initial conditions are *a priori* unidentifiable as CD-resilient or CD-susceptible, this prompts a clinically relevant caution of whether some donor’s microbiome will be beneficial or detrimental to another’s microbiome.

In a recent experimental study by Buffie *et al.* [13] CD-vulnerable mice exposed to CD were given transplants consisting of a known microbial composition, and the transplant efficacy for each composition was measured. Our work on simulated transplants, which resembles the experimental study, provides context and explanation for the mechanism of the experimental transplants. In conjunction, simulated and experimental transplants could direct the development of model-guided and experimentally-validated “designer” transplants.

### Simulated antibiotic dosing

Antibiotic administration has traditionally been the standard approach to fight infection, but antibiotics have struggled to control CD infection: CDI has a recurrence rate of 30-65% following antibiotic treatment, while fecal transplantation has cure rates upwards of 90% [33]. Nonetheless, the Society for Healthcare Epidemiology of America (SHEA) and the Infectious Diseases Society of America (IDSA) jointly recommend treating CDI with antibiotics— often vancomycin— administered in one of three dosing regimens: a constant dosing regimen, a pulsed dosing regimen, or a tapered dosing regimen [16]. Other studies have found that vancomycin administered in tapered or pulsed doses reduced the likelihood of recurrent infections of CD, compared with treatment at a constant dosage [34]. Our model, which allows arbitrary control over the dosing schedule and concentration *u*(*t*), provides a computational framework on which we can compare the efficacy of different dosing schedules: our implementations of the three dosing regimens are plotted in S3 Fig.

Over short time scales of 1-2 days we found that given the same total amount of antibiotic, the rate at which antibiotics were administered (e.g. .5 doses for 2 days vs. 2 doses for.5 days) did not affect the eventual steady state. Over longer time scales of around 2 weeks, we observed similar behavior— the model does not capture long-term differences between different dosing regimens as long as the total amount of administered antibiotic is the same, reflecting that the time-scale for microbial growth is longer than the period over which antibiotics are typically administrated.

In modeling the different dosing regimens, we are faced with one main complication: only one antibiotic, clindamycin, was fit in [11], and furthermore clindamycin was acting to *induce* CDI rather than halt it. The antibiotic efficacy parameter ***ε*** therefore does not serve as a realistic proxy for vancomycin or metronidazole, antibiotics which are used to eliminate CD [34]. To simulate the effect of an antibiotic which eliminates CD, we introduce an artificial “targeted antibiotic” $\tilde{\varepsilon }$, which by construction only inhibits CD; specifically, ${\tilde{\varepsilon }}_{c}=-1$ and ${\tilde{\varepsilon }}_{i}=0\;\text{for}\;i\neq c$.

**Fig 7:**
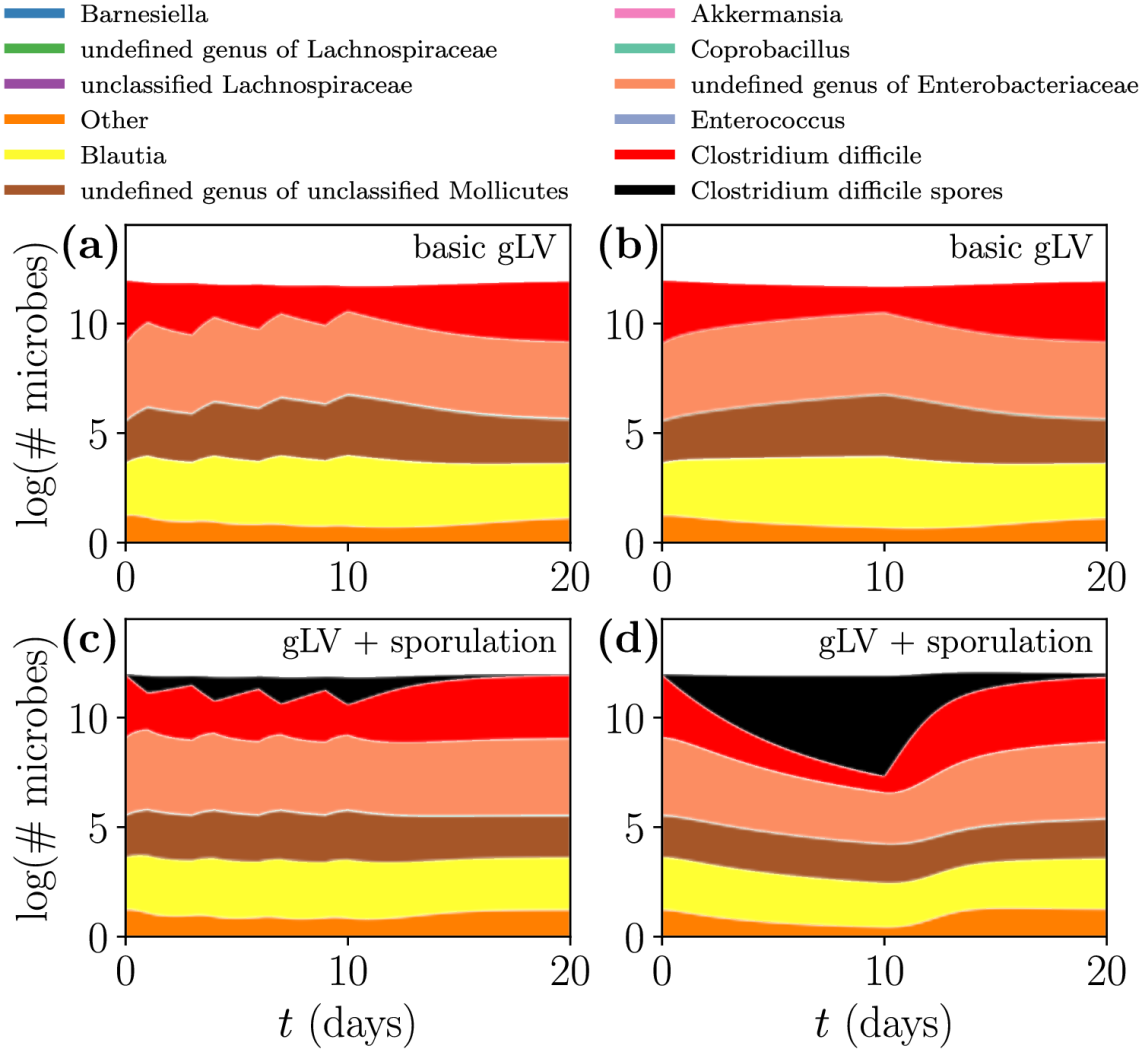
Different antibiotic treatment regimens influence transient CD dynamics when considering sporulation. All scenarios start from infected steady state D. Over 10 days, the same volume of “targeted” antibiotic is administered via a pulsed (a,c) or constant (b,d) dosing regimen. The top panels use the original gLV model Eq (1) while the bottom panels use the sporulation gLV model Eq (3). For details about the parameters used in this figure, refer to Table A of S1 Appendix.

Even with this targeted antibiotic our model does not capture significant differences between the treatment regimens, which is contrary to the clinical recommendation that pulsed or tapered dosing be preferred over constant dosing [16]. In Fig 7a and 7b we administer the same amount of targeted antibiotic via constant and pulsed dosing to the CD-infected steady state and find that the two dosing regimens produce near-identical microbe trajectories (a similar result, shown in S4 Fig, was found with tapered dosing). We propose sporulation (which acts on a much shorter time-scale) as a biologically relevant mechanism that could explain this inconsistency.

### Sporulation

In considering the model for sporulation detailed in Eq (3), the steady state analysis we previously performed is still relevant since all steady states will eventually be spore-free— we assume that the antibiotics will eventually cease, so the sporulation term of Eq (3) will eventually decay exponentially. However, the naïve expectation that including sporulation would make CD-infected steady states more common is incorrect; once again, due to the interactions between CD and other background microbes (mediated by the interaction matrix *M*), the presence of CD encourages growth of the microbes that populate the infection-free steady state, and so increasing the prevalence of CD through sporulation only entrenches the non-infective steady state. Since the steady states and phase diagrams are mostly unchanged by the inclusion of sporulation, we concentrate on the dynamics of CD and CD spores on shorter time scales.

In Fig 7 we compare the effects of the standard gLV model Eq (1) (top panels) and the sporulation model Eq (3) (bottom panels) under constant and pulsed antibiotic dosing regimens. Here, we use the targeted antibiotic previously described and apply all treatments to the CD-infected steady state D. Sporulation causes spores to form as antibiotics are administered, and germinate once the antibiotics cease, which is on display in the pulsed dosing regimen scenario of Fig 7c. After targeted antibiotic administration CD recovers slightly more quickly with sporulation than without, and we interpret this expedited resurgence as a more robust CDI. For details about the parameters used in Fig 7, refer to Table A of S1 Appendix.

While many of the in-host dynamics and the biological mechanisms that underlie CD sporulation and germination remain under active investigation, studies have identified that both spores and vegetative CD colonize and persist in the gut [35], and other studies have discerned the role of bile acids in promoting spore germination [36]. Our model does not allow for the long-term establishment of spores because we assume that germination always occurs in the absence of antibiotics, and we include no mechanism for germination induced by bile acids. However, more detailed sporulation models (e.g. models that include bile acid-induced germination) may extend our basic model to build upon the qualitative features of CDI it possesses.

We emphasize that sporulation is simply a proposed biological mechanism that would modify the model’s predictions to better match clinical observations, and so these results should be interpreted in a qualitative manner. However, by including sporulation we regain (at least for short time scales) the clinically expected result [16] that pulsed dosing is more effective than constant dosing at eliminating CD— comparing the top panels with the bottom panels of Fig 7 indicates that a pulsed dosing regimen dramatically reduces the buildup of CD spores compared to constant dosing.

### Mutation

The mechanism of mutation, introduced in Eq (4), introduces new unconstrained parameters for the mutation rate *k* as well as for pairwise interactions *M*_*im*_ and *M*_*mi*_. Here we identify an intuitive parameter choice that reflects the underlying biology, discuss the resultant steady states, and then demonstrate the effects of mutation on transient microbe dynamics.

Antibiotic-resistant mutations typically incur a fitness cost in the absence of antibiotics since resources are being allocated for defense against antibiotics rather than growth [37–39], so we choose *μ*_*m*_ = .9*μ*_*c*_ < *μ*_*c*_. Our choice of *M* assumes that the background microbes interact with mutant and native types identically (i.e. *M*_*mi*_ = *M*_*ci*_ and *M*_*im*_ = *M*_*ic*_ for *i* ≠ *c*, *m*). In real systems, the mutation rate *k* is variable and depends on factors including the concentration of antibiotic, the type of antibiotic, the native strain type, and other environmental pressures. In our model we approximate the mutation rate as a constant *k* = 2 * 10^−6^ (in units of 1/day), a choice which is in the range of measured mutation rates of some bacteria [40], but for our purposes mostly serves to highlight the effects of mutation. For details about the parameters used in our simulations of the mutation model, refer to Table A of S1 Appendix.

**Fig 8:**
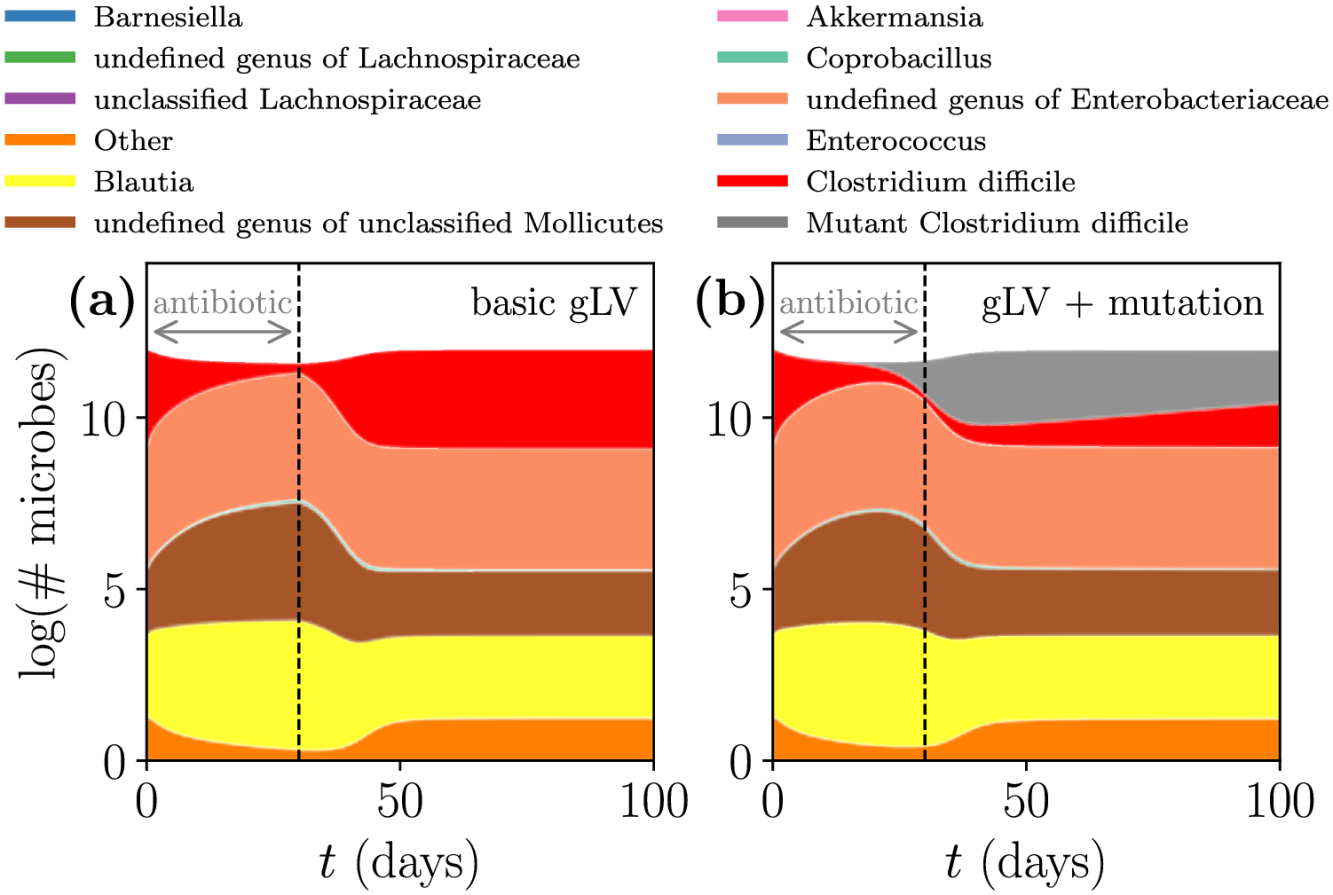
Antibiotic-resistant mutation improves CD resilience to antibiotics in transient dynamics. These simulations are identical except that (a) uses the original gLV model Eq (1) while (b) uses the mutation gLV model Eq (4). This scenario starts from the CD-infected steady state D and administers an idealized “targeted” antibiotic (that only inhibits CD) for 30 days. For details about the parameters and initial conditions used in this figure, refer to Table A of S1 Appendix.

Due to our parameter choices the steady states of the background microbes are largely unchanged between the mutation model and the basic model (the CD-infected steady states of the standard and mutation models are explicitly compared in Table B of S1 Appendix), but the transient dynamics shown in Fig 8 differ. In these plots the same amount of targeted antibiotic is applied to the same initial state, but Fig 8a uses the standard gLV model Eq (1) while Fig 8b uses the modified mutant gLV model Eq (4). The targeted antibiotic severely inhibits CD in the standard gLV model, but in the mutation model the antibiotic-resistant mutant compensates for the antibiotics and reinforces the colonization of CD despite the antibiotic pressures.

At the scale of a single bacterium experiments now track the growth and decline of individual lineages of bacteria when confronted with antibiotics [41], and at larger scales experiments track the spread and fixation of mutations across an entire bacterial community [42]. Since the gLV model considers populations of bacteria rather than individual cells, the individual lineages cannot be resolved. However, our model does capture the tendency of microbes with a selective advantage to outcompete microbes with lower fitness (in our case, CD mutants outcompete native CD in the presence of antibiotics), and these simulations resemble the selective sweeps found in experimental data [42].

Existing mutation models have studied native and mutant strains of bacteria in isolation, but by embedding mutation within a gLV framework we can probe the complex behaviors of mutant strains within a microbial consortia. Accordingly, the wealth of behaviors present in the simpler mutation models [28] may be observed within the gLV model with mutation Eq (4). This comprehensive and community-level view is essential in identifying, understanding, and resolving the role of antibiotic-resistant mutants in disease.

## Discussion

### Application of framework for experimental explanation, model validation, and suggestion of model-motivated experiments

A study by Buffie *et al.* [13] follows the modeling method of Stein *et al.* [11] and fits a gLV model to both mouse and human experimental time-series data in order to predict the growth of CD following antibiotic administration. In this study they identify the microbes anticorrelated with CDI in experimental data as well as the microbes that most inhibit the growth of CD according to the interaction matrix *M* of the gLV model. They create and administer transplants made up of a subset of these identified microbes: four transplants consist of individual microbes in isolation while another consists of a combination of all four microbes.

Buffie *et al.* [13] find that of the four transplants made up of isolated microbes only one microbe is effective at curing CDI, despite the fact that the other three microbes were *a priori* supposed to inhibit CD. We provide two explanations for their findings, motivated by the results of our paper: (1) the ability of CD-resilient transplants to confer CD-colonization resistance is largely variable and depends on the transplant composition (e.g. the variation in transplant efficacies between Fig 5 and S2 Fig), and (2) inhibiting the growth of CD does not necessarily inhibit CD-infected steady states, since the presence of CD inhibits some of the microbes that populate the CD-infected steady state. By applying the results of our simulations to microbiome transplant experiments, we can offer a computational context for experimental results.

Many of the microbes identified by Buffie *et al.* [13] as potential transplants were of the genus *Clostridium*; in fact, the only isolated-microbe transplant that was effective in curing CDI was *Clostridium scindens*. If we resolve only to the genus level (as assumed when constructing the gLV model in Stein *et al.* [11]), this experimental result is consistent with our own transplant simulations in which a transplant made up of the CD-resilient IC 8 was significantly more effective with CD than without (Fig 5). Hence, the seemingly contradictory computational result— that the presence of CD inhibits CD-infected steady states— is validated by experiment.

Finally, we can formulate experimental questions that are couched in our computational framework. Our results point to the importance of timing when administering microbial transplants, an area that is mostly unexplored both experimentally and therapeutically, and experiments could elucidate how the the timing of transplants effects their efficacy. While Buffie *et al.* [13] inferred microbial interactions from a gLV model, when predicting the CD-inhibiting microbes their analysis did not include dynamic simulations; applying our method of simulated transplants to such experiments could inform the selection of “personalized” transplants, and the corresponding experiments could then be used to inform the model, the model’s limitations, and additional experiments.

### Unidentifiability of beneficial bacterial communities

In this paper, the principle driver of CDI was whether a given microbial composition was CD-resilient or CD-susceptible: for example, when administering a fecal transplant, the effectiveness of the treatment depended on the properties of the donor’s microbiome. In general these properties are unknown *a priori*, so picking the right donor is a gamble. In clinical practice, the screening process for potential fecal donors consists primarily of avoiding those with impaired microbiomes (e.g. due to recent antibiotic therapy) or poor health, with only about 10% of prospective donors being accepted [43, 44]. While fecal transplantation has been more successful at curing CDI than traditional antibiotic treatments [45], predictive models are not currently being implemented to quantitatively select an optimal donor. Eventually, predictive models could allow for “designer” fecal transplants that are engineered to optimally confer colonization resistance. Until donor selection methods consist of searching for optimal donors rather than excluding diseased donors, our model warns that donor selection— even of seemingly healthy donors— can have unexpected consequences.

### Pharmacokinetic and pharmacodynamic approximations

In this paper, we follow Stein *et al.* [11] and model the pharmacokinetics (the in-host concentration of the antibiotic *u*(*t*)) as a pulse. In reality, clindamycin pharmacokinetics are characterized by an initial spike in the in-host antibiotic concentration, after which antibiotics are cleared from the system (driven by uptake and deterioration of the antibiotic) with a half-life of approximately 4 hours [46]. However, in our simulations we found that over short durations (1-14 days) it is the total amount of administered antibiotic that determines the long-term dynamics of Eq (1) rather than the shape of the dosing regimen *u*(*t*) (meaning that administering.5 doses for 2 days leads to the same outcome as administering 2 doses for.5 days). This insensitivity to the form of *u*(*t*) justifies our simplified pharmacokinetic form.

Additionally, we model the pharmacodynamics (the microbial response or killing rate due to antibiotics) as a linear response −*ε*_*i*_ *u*(*t*), while more realistic models use a saturating Hill function [47, 48]. However, we only use one antibiotic concentration for each simulation, corresponding to one killing rate for each simulation. For any killing rate in the range of the saturating Hill function, one may find an effective antibiotic concentration that achieves this killing rate via either the linear response or by the saturating Hill function. Since both the linear response and the saturating Hill function are monotonic, there is a nonlinear scaling for *u*(*t*) between the two response functions, meaning that our results— acquired with the linear response function— may be extended to a model that uses a saturating Hill function as long as the antibiotic scaling is observed (e.g. for the phase diagram of Fig 5, stretch the antibiotic axis). Since a linear antibiotic response qualitatively captures the same long-term dynamics that a saturating Hill function would, we are justified in using a simplified pharmacodynamic model.

### Limitations of the gLV model

The gLV model idealizes interspecies interaction, and this simplification imposes limitations on our framework. The gLV model does not explicitly model *why* populations grow or decay (due to the underlying resource excesses or limitations) [49], and populations are assumed to respond instantly to changes in other populations, failing to account for the time required to respond to change [50]. The number of parameters required for a gLV model scales as *N*^2^ for *N* species, and even with high-throughput sequencing, the number of data points per parameter is still low (e.g. roughly 5 data points per parameter in [11]). Despite these drawbacks, gLV models are commonly implemented to describe microbial growth [14] since they are predictive, manipulable, and often capture the qualitative characteristics of microbial consortia. Our framework attempts to resolve some of these limitations by treating the gLV model as a base model, then offering extensions to the model that incorporate nongeneric and mechanistic features in order to more accurately portray microbial growth.

### Analytic concerns of parameter fitting

There are many techniques that fit parameters to data [51, 52], but it is difficult to know that these fitted parameters are indeed the true parameters. Stein *et al.* [11] fit the parameters used in this paper with regularized linear regression with a Tikhonov regularization, but other fitting methods exist, such as LIMITs [53], a software specifically designed for fitting microbial time-series data to a gLV model. Analytically, there are sufficient conditions on the model parameters that ensure the Lyapunov stability of fixed points of generalized Lotka-Volterra systems [9], but the fitted parameter values in this paper do not satisfy all of these conditions. Leveraging fitting methods to simultaneously fit parameters to data while maintaining the analytic properties that ensure stability would alleviate the potential for non-biological divergences in microbe count (divergences which are not impossible in the given system since *M* has a single positive eigenvalue). Regardless of this possibility, no unstable behavior was observed in any of the simulations run for this paper, perhaps due to the relatively few symbiotic relationships.

### Combining gLV and SIR techniques

In this paper we fuse standard SIR techniques with the gLV model, thereby introducing specific mechanisms for sporulation and mutation. In this way, our framework allows for non-generic attributes of populations to be captured and simulated, and the resulting analyses provide qualitative insights into different mechanisms. Effectively, this allows for the entire family of SIR methods to be used in conjunction with the gLV model.

### Conclusion

As the era of personalized medicine approaches, there is a growing need for accurate computational models that reflect human biology and can predict the progress of disease. This pursuit will be aided by the availability of “big data” in medicine, but this data needs to be harnessed in a useful way. This paper addresses initial steps in developing these computation models by constructing a framework at the interface between computational models and clinical therapies. This modular framework allows for “plug-and-play” implementations of clinical techniques and observed phenomena: in this paper, we implement fecal transplant therapy, antibiotic treatment regimens, sporulation, and mutation.

Our in-silico implementations of clinical treatments were mostly congruent with the actual clinical realizations— there exist initial conditions that become susceptible to CD after exposure to antibiotics; administration of a fecal transplant can halt CDI; and (once sporulation is included) pulsed dosing is more effective at eliminating CD than constant dosing, though fecal transplants are more effective than antibiotic administration in the long run. Introducing mechanisms for antibiotic-resistant mutations and sporulation strengthens the resilience of CD to remedial treatments. In all, this framework captures the intention and qualitatively the results of real-world clinical techniques.

There are many avenues stemming from this framework that may be explored in the future, including research into “designer transplants” or of bile acid-mediated germination of CD spores. Eventually this framework could be used to suggest clinical practices, but first more experiments, better data, and novel modeling are needed. As we recognize the advancement of gene sequencing in the past few years, it is not inconceivable that user-specific personalized medicine programs, built upon mathematical models of human health, will be accessible in the future.

## Supporting information

S1 Fig Microbial composition of reachable steady states. Under the gLV model Eq (1) and for all experimentally measured initial conditions, all treatment scenarios tested in this paper result in one of the steady states A-E. To find which steady state a given treatment scenario causes, refer to Fig 4. Note that while steady states A and D appear indistinguishable in this plot, their compositions do vary slightly. The microbial compositions of each steady state are explicitly given in Table B of S1 Appendix.
(TIF)

S2 Fig Different CD-resilient initial conditions have different transplant efficacies. Starting from the CD-fragile initial condition, antibiotics of varying antibiotic concentration are administered on day 0, and the system is exposed to CD on day 1. Then, a transplant made up of the CD-resilient initial condition 2, to contrast Fig 5 which used IC 8, is infused on day *d*. Note that for a transplant from this donor IC 2 to be effective, the relative transplant size needs to be much greater than when using IC 8. The *infected* region corresponds to infected steady state D, and the *uninfected* region corresponds to uninfected steady state E. A relative transplant size of 1 corresponds to a transplant that has the same size as the initial condition that the transplant was derived from.
(TIF)

S3 Fig Antibiotic dosing regimens. We consider three types of antibiotic treatment *u*(*t*), displayed here, in the gLV model Eq (1). These dosing regimens— constant, tapered, and pulsed— are common in clinical practice. In this example, 2 doses are administered over 10 days.
(TIF)

S4 Fig Tapered and constant antibiotic treatment regimens produce qualitatively similar microbial trajectories. Both scenarios start from infected steady state D. Over 10 days, the same volume of “targeted” antibiotic is administered via a tapered (a) or constant (b) dosing regimen, with microbial trajectories evolving according to the original gLV model Eq (1). The parameters used in this figure are the same as in Fig 7a and 7b.
(TIF)

S1 Appendix Supplementary tables. Parameter values used for simulations (Table A) and microbial compositions of initial conditions and steady states (Table B).
(PDF)
