# Supplementary figures and images for: In silico analysis of antibiotic-induced Clostridium difficile infection: Remediation techniques and biological adaptations
Source: PLoS Comput Biol. 2018 Feb 16;14(2):e1006001. doi: 10.1371/journal.pcbi.1006001 (PMC5833281; doi:10.1371/journal.pcbi.1006001)

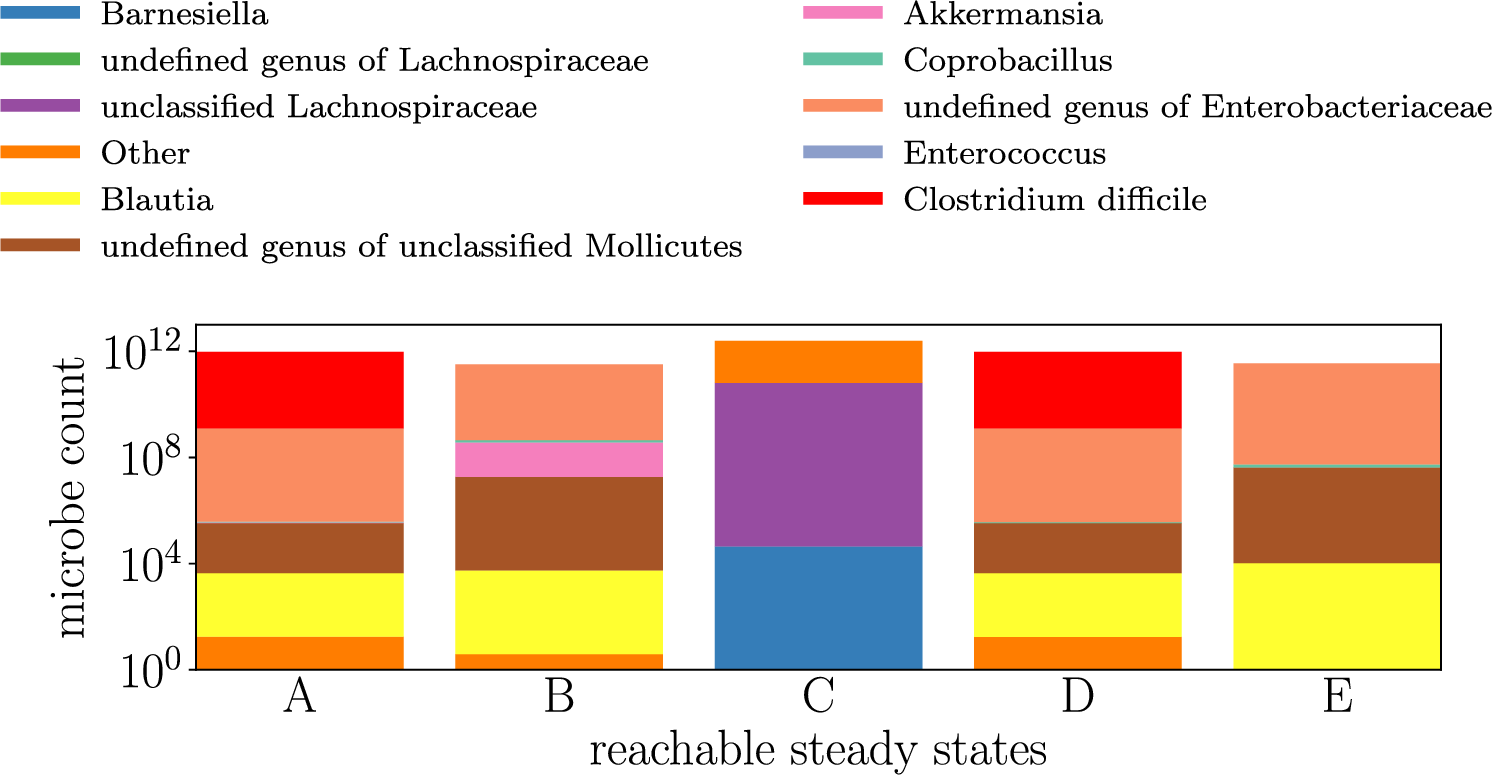

Supplement: S1 Fig — Under the gLV model Eq (1) and for all experimentally measured initial conditions, all treatment scenarios tested in this paper result in one of the steady states A-E. To find which steady state a given treatment scenario causes, refer to Fig 4. Note that while steady states A and D appear indistinguishable in this plot, their compositions do vary slightly. The microbial compositions of each steady state are explicitly given in Table B of S1 Appendix. (TIF) [file pcbi.1006001.s001.tif]

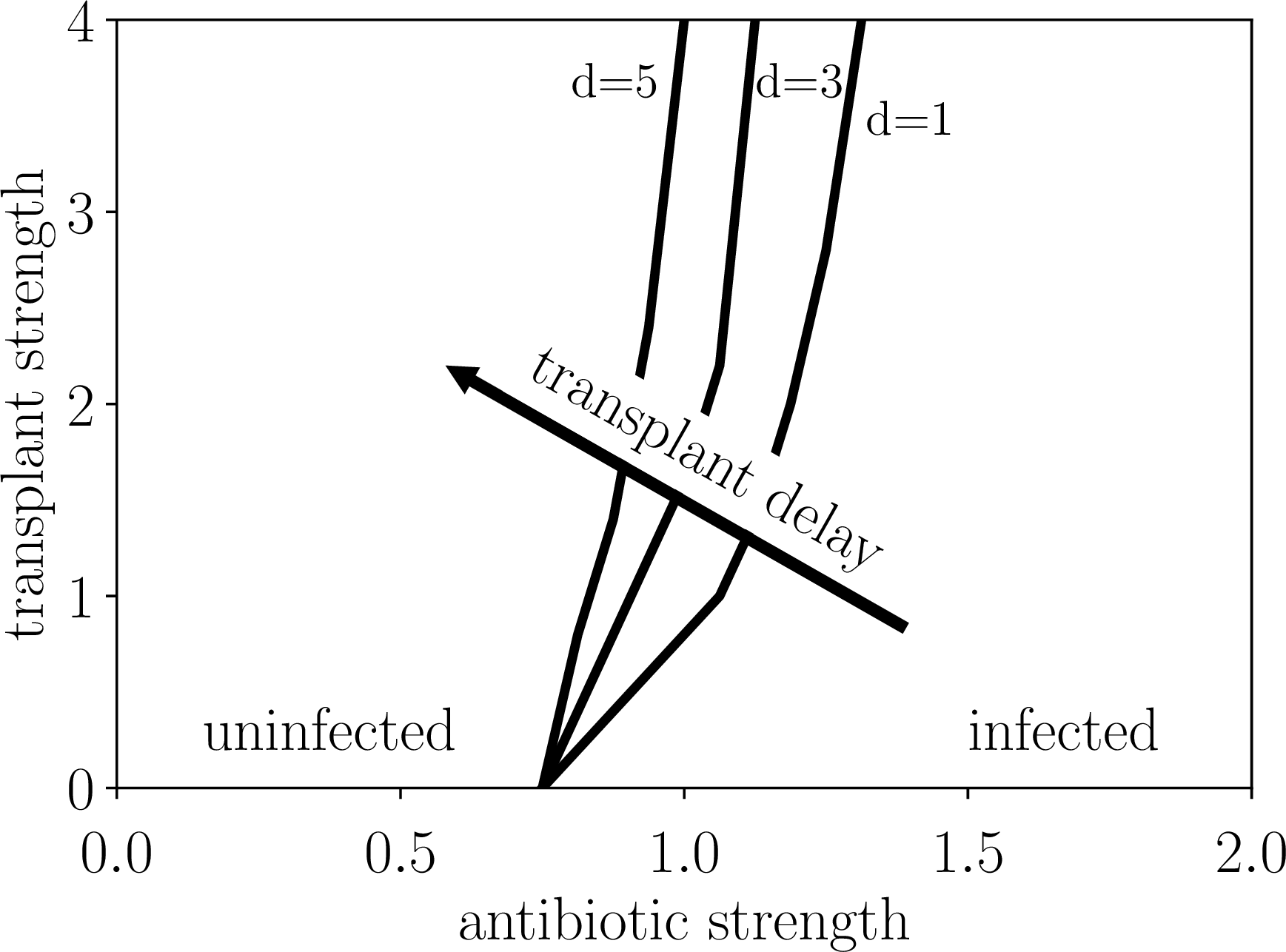

Supplement: S2 Fig — Starting from the CD-fragile initial condition, antibiotics of varying antibiotic concentration are administered on day 0, and the system is exposed to CD on day 1. Then, a transplant made up of the CD-resilient initial condition 2, to contrast Fig 5 which used IC 8, is infused on day d. Note that for a transplant from this donor IC 2 to be effective, the relative transplant size needs to be much greater than when using IC 8. The infected region corresponds to infected steady state D, and the uninfected region corresponds to uninfected steady state E. A relative transplant size of 1 corresponds to a transplant that has the same size as the initial condition that the transplant was derived from. (TIF) [file pcbi.1006001.s002.tif]

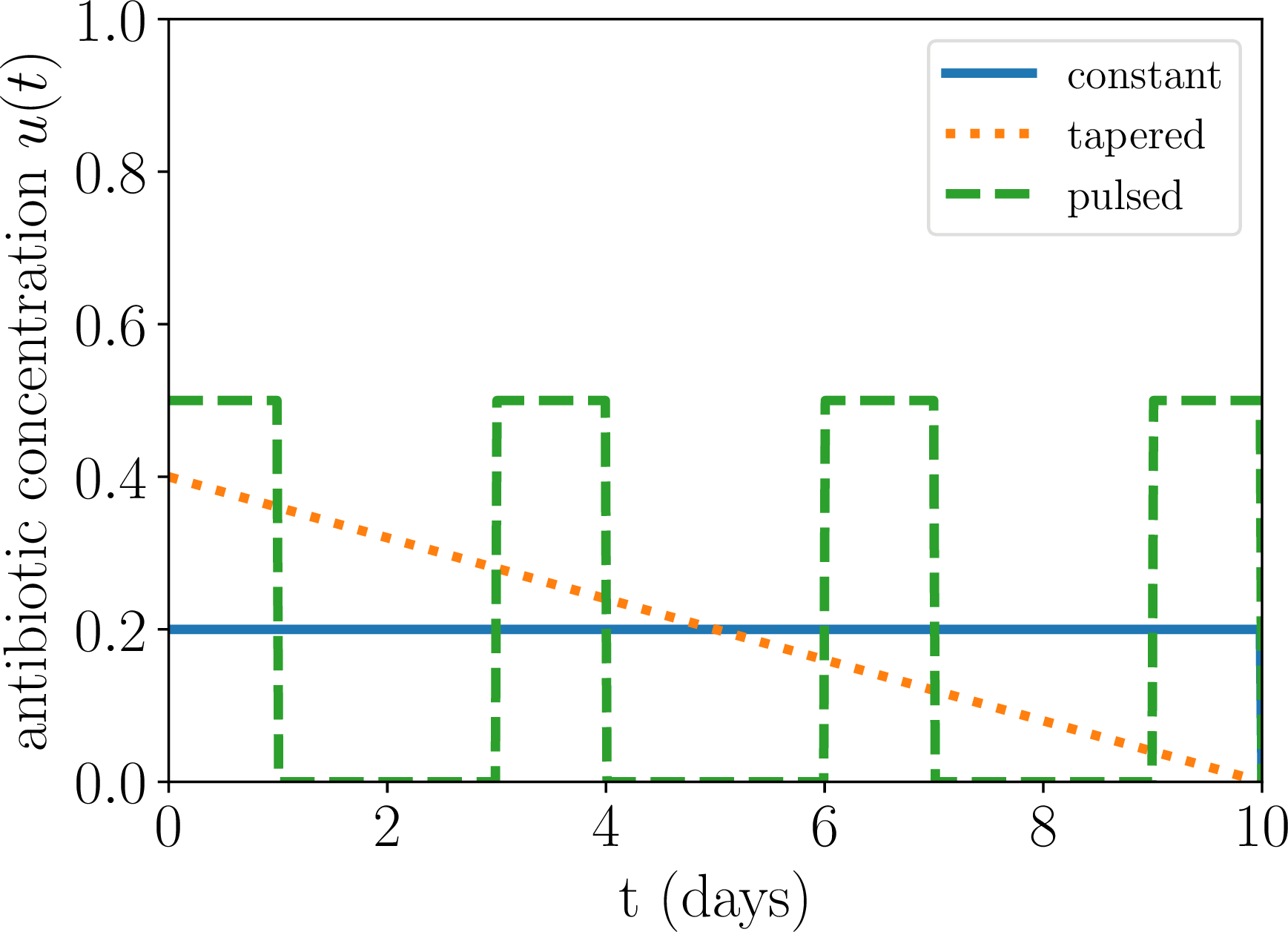

Supplement: S3 Fig — We consider three types of antibiotic treatment u(t), displayed here, in the gLV model Eq (1). These dosing regimens— constant, tapered, and pulsed— are common in clinical practice. In this example, 2 doses are administered over 10 days. (TIF) [file pcbi.1006001.s003.tif]

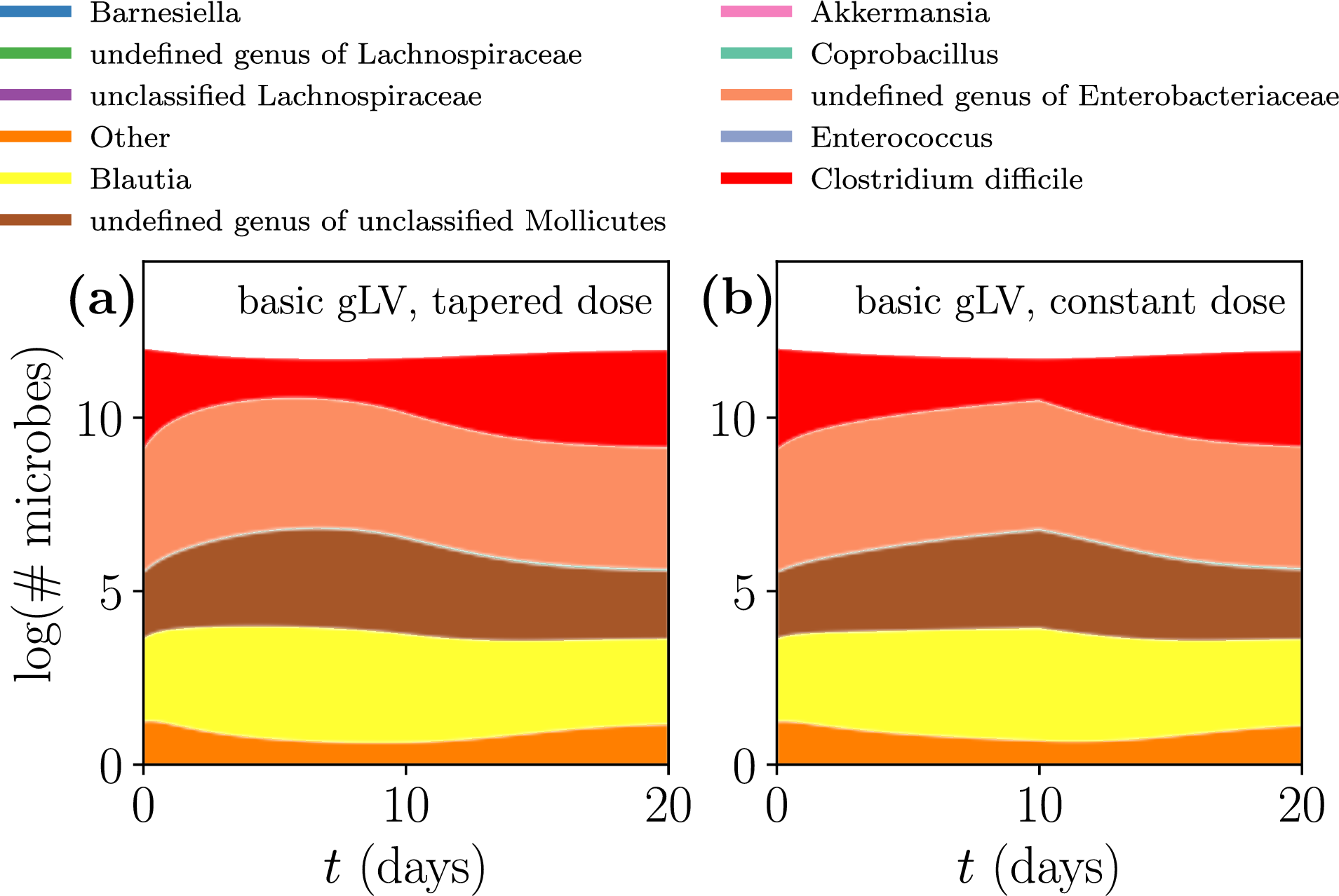

Supplement: S4 Fig — Both scenarios start from infected steady state D. Over 10 days, the same volume of “targeted” antibiotic is administered via a tapered (a) or constant (b) dosing regimen, with microbial trajectories evolving according to the original gLV model Eq (1). The parameters used in this figure are the same as in Fig 7a and 7b. (TIF) [file pcbi.1006001.s004.tif]
