# Supplementary material for: In silico analysis of antibiotic-induced Clostridium difficile infection: Remediation techniques and biological adaptations
Source: PLoS Comput Biol. 2018 Feb 16;14(2):e1006001. doi: 10.1371/journal.pcbi.1006001 (PMC5833281; doi:10.1371/journal.pcbi.1006001)
Supplement: S1 Appendix — Parameter values used for simulations (Table A) and microbial compositions of initial conditions and steady states (Table B). (PDF) [file pcbi.1006001.s005.pdf]

In silico analysis of antibiotic-induced *C. difficile* infection: remediation techniques and biological adaptations

S1 Appendix

Eric Jones* and Jean Carlson

Department of Physics, University of California at Santa Barbara, Santa Barbara, California, United States of America

* ewj@physics.ucsb.edu

Table A. Parameter values for simulations. The nine experimental initial conditions (labeled "IC 1" through "IC 9") are from the supplementary information of Stein *et al.* [1] in `Dataset_S2.xlsx` tab `Y`, and the initial conditions are also explicitly given in Table B of S1 Appendix. The parameters M, $\boldsymbol{\mu}$, and $\boldsymbol{\varepsilon}$ are also from the supplementary information of Stein *et al.* in `Dataset_S2.xlsx` tab `MmuE`. All simulations of the basic gLV model Eq (1), unless otherwise noted, use these parameters. We denote a constant antibiotic treatment of concentration c that starts on day a and ends on day b by by $u(c, [a, b])$. "CD inoculation" consists of the introduction of 10^{-10} nondimensionalized units of CD (equivalent to 10 rRNAcopies/cm^3 of CD in dimensionalized units; for nondimensionalization details see Stein *et al.* [1]). Unless otherwise specified, simulations that attain a steady state are run for 1000 days.

Fig #	Model	Parameters and initial conditions
Fig 1	Eq (1) (basic gLV)	The food web only displays interactions that satisfy $\lvert M_{ij}\rvert > 0.30$ for clarity.
Fig 2	Eq (1) (basic gLV)	Simulations are run on all initial conditions. For each initial conditions, the antibiotic treatment is $u(1, [0, 1])$, and CD inoculation occurs on day 10.
Fig 3	Eq (1) (basic gLV)	Four different treatments scenarios are applied to IC 5: in Fig 3a no interventions occur; in Fig 3b the antibiotic treatment is $u(1, [0, 1])$ with no CD inoculation; in Fig 3c the antibiotic treatment is $u(0.5, [0, 1])$ followed by CD inoculation on day 10; and in Fig 3d the antibiotic treatment is $u(1, [0, 1])$ followed by CD inoculation on day 10.
Fig 4	Eq (1) (basic gLV)	For each IC, Eq (1) is simulated for a range of antibiotic concentrations (with antibiotic treatments ranging from $u(0, [0, 1])$ to $u(2, [0, 1])$ with an antibiotic concentration step size of 0.1) and with or without CD inoculation on day 10. For each choice of IC, antibiotic concentration, and CD exposure, the system is simulated until it reaches a steady state. The Shannon diversity of each steady state is calculated (equal to $-\sum_{i=1}^{N} p_i \log p_i$, where p_i is the proportion of the steady state that is microbe i), and this Shannon diversity effectively hashes the steady state into a unique number. Distinct steady states (with different diversities) are then delineated and enumerated, and the resultant phase diagram of each IC is plotted.
Fig 5	Eq (1) (basic gLV)	For IC 5, Eq (1) is simulated for a range of antibiotic concentrations (with antibiotic treatments ranging from $u(0, [0, 1])$ to $u(2, [0, 1])$ with an antibiotic concentration step size of 0.0625), over a range of transplant sizes (transplants are derived from IC 8, and relative transplant sizes vary from 0 to 1 with a step size of .05; the resultant transplant is IC 8 scaled by the relative transplant size), and over a range of transplant timings (the transplant is administered on day 0, 1, 7, 10, 14, or 30). For all parameter choices, the system is inoculated with CD on day 1. The resulting steady states— one CD-infected and the other CD-uninfected— are distinguished in the same way as in Fig 4. Simulations are run for 2000 days.
Fig 6	Eq (1) (basic gLV)	IC 5 is simulated with an antibiotic treatment $u(1, [0, 1])$, CD inocuation on day 10, and a transplant derived from IC 8 of relative transplant size 0.1 administered either on day 1 (Fig 6a) or on day 7 (Fig 6b).
Fig 7	Eq (1) (basic gLV), Eq (4) (sporulation)	The initial condition is the CD-infected SS, explicitly given in Table B of S1 Appendix, which may be obtained by simulating IC 5 with antibiotic treatment $u(1, [0, 1])$ and with CD inoculation on day 10. This CD-infected steady state is the same in both gLV models. In all subfigures, 3 doses of targeted antibiotics are administered over 10 days: in Figures 7a and 7c, the antibiotic is adminstered in a pulsed regimen, described in S2 Fig, and in Figures 7b and 7d the antibiotic is administered at a constant rate corresponding to an antibiotic treatment $u(.3, [0, 10])$. Figures 7a and 7b use Eq (4) but use the targeted antibiotic $\tilde{\varepsilon}$ described in the Simulated Antibiotic Dosing section of the main text. Figures 7c and 7d use the sporulation model Eq (4) with the additional sporulation parameters $\alpha = .312$, $\beta = .312$, and $u_{spor} = 0.05$.
Fig 8	Eq (1) (basic gLV), Eq (5) (mutation)	In Fig 8a the initial condition is the CD-infected SS as described in Fig 7. In Fig 8b the initial condition is the mutation model version of the CD-infected SS, which is obtained in the same way as in Fig 7 except that the mutation model Eq (5) is used rather than the basic model Eq (1); an explicit comparison of these steady states is in Table B of S1 Appendix. The targeted antibiotic $\tilde{\varepsilon}$ is used in both subfigures with the antibiotic treatment $u(0.3, [0, 30])$. In Fig 8b, the additional mutation parameters are $\mu_m = .9\mu_c$; $M_{im} = M_{ic}$ for $i \neq c, m$; $M_{mi} = M_{ci}$ for $i \neq c, m$; $M_{cm} = M_{cc}$; $M_{mc} = -.04$; $M_{mm} = -.05$; and $k = 2 * 10^{-6}$.

Table B. Microbial compositions of initial conditions and steady states. Microbial compositions of the nine experimental initial conditions (top) measured by Stein *et al.* [1], and of the obtained steady states (bottom) described in Figs 4 and 8. The provided values are in nondimensionalized units, but 1 in nondimensionalized units corresponds to 10^{11} rRNAcopies/cm^3 in dimensionful units (for details refer to Stein *et al.* [1]).

**Initial conditions:**	**IC 1**	**IC 2**	**IC 3**	**IC 4**	**IC 5**	**IC 6**	**IC 7**	**IC 8**	**IC 9**
Barnesiella	0.0927	1.7504	0.0098	0.0674	2.7665	1.7757	0.2364	2.5396	1.397
undefined genus of Lachnospiraceae	0.2196	2.6576	0.0074	0.0869	3.0209	0.0096	0.0037	2.4263	1.0672
unclassified Lachnospiraceae	0.0325	0.6562	0.0036	0.0209	0.5964	0.0003	0.0002	0.6845	0.2005
Other	0.1114	0.9745	0.0116	0.0331	1.931	0.0754	0.7666	1.8417	0.6187
Blautia	0.0009	0.0066	0.0001	0.0005	0.0097	0.4832	0.0002	0.0085	0.0045
undefined genus of unclassified Mollicutes	0.0014	0.0115	0.0002	0.0004	0.0083	0.0912	0.0004	0.0122	0.002
Akkermansia	0.0331	0	0	0.0006	0	0.0007	0.0007	0	0.0003
Coprobacillus	0.0001	0.0082	0	0.0002	0.0097	0.0005	0	0.0085	0.0035
undefined genus of Enterobacteriaceae	0	0	0	0	0.0012	0.2332	0.0029	0	0.0003
Enterococcus	0	0	0	0	0	0.0001	0.0007	0	0.0001
Clostridium difficile	0	0	0	0	0	0	0	0.001	0
Total steady state size	0.4918	6.0649	0.0326	0.21	8.3438	2.6699	1.0117	7.5223	3.2942

**Steady states:**	**A (infected)**	**B (susceptible)**	**C (resilient)**	**D (infected)**	**E (susceptible)**	**Mutant SS (infected)**
Barnesiella	0	0	9.299	0	0	0
undefined genus of Lachnospiraceae	0	0	0	0	0	0
unclassified Lachnospiraceae	0	0	12.3085	0	0	0
Other	0.9844	0.1667	3.1627	0.9839	0.006	0.9507
Blautia	1.882	0.8871	0	1.9081	1.2284	1.8852
undefined genus of unclassified Mollicutes	1.4871	0.9898	0	1.4974	1.1055	1.4841
Akkermansia	0.0214	0.3664	0	0	0	0
Coprobacillus	0.0237	0.0234	0	0.0243	0.0352	0.0249
undefined genus of Enterobacteriaceae	2.7709	0.8046	0	2.807	1.1694	2.7514
Enterococcus	0	0	0	0	0	0
Clostridium difficile	2.2651	0	0	2.2858	0	1.0022
Mutant Clostridium difficile	—	—	—	—	—	1.207
Total microbe count	9.4347	3.238	24.7702	9.5065	3.5445	9.3054

References

1. Stein RR, Bucci V, Toussaint NC, Buffie CG, Rätsch G, Pamer EG, et al. Ecological Modeling from Time-Series Inference: Insight into Dynamics and Stability of Intestinal Microbiota. PLoS Comput Biol. 2013;9(12):1–11. doi:10.1371/journal.pcbi.1003388.
